# Strategy for enhanced production of A40926B0 in *Nonomuraea gerenzanensis* using an efficient CRISPR/AsCas12f1 system

**DOI:** 10.1016/j.synbio.2026.08.006

**Published:** 2026-08-24

**Authors:** Xiaoru Wang, Hongmei Zhai, Dandan Gu, Yongchao Wang, Jiali Gao, Luxi Du, Liuxu Zhuo, Xiaobing Li, Yu Wang

**Affiliations:** a College of Chemical Engineering, Shijiazhuang University, Shijiazhuang, Hebei Province, 050035, China; b Hebei Provincial University Microbiology Pharmaceutical Application Technology Research and Development Center, Shijiazhuang, 050035, China; c Hebei Key Laboratory of Pharmaceutical Polymer Excipients, Shijiazhuang, 050035, China; d Hebei International Joint Research Center for Biopharmaceutical, Shijiazhuang University, Shijiazhuang, Hebei Province, 050035, China

**Keywords:** *Nonomuraea gerenzanensis*, A40926B0, Dalbavancin, CRISPR/AsCas12f1, Metabolic engineering, Shikimate pathway, Branched-chain fatty acid

## Abstract

The global emergence of vancomycin-resistant Gram-positive pathogens underscores the urgent need for efficient production of novel lipoglycopeptide antibiotics. Dalbavancin, a last-resort therapeutic agent, relies on its key biosynthetic precursor A40926B0, whose industrial manufacture is severely limited by the low yield of wild-type *Nonomuraea gerenzanensis* and inefficient genetic tools for this rare actinomycete. Here, we developed a high-efficiency CRISPR/AsCas12f1 genome editing system and applied systematic metabolic engineering to boost A40926B0 biosynthesis. First, conjugation conditions were optimized to elevate the transfer efficiency in *N. gerenzanensis* D11. The hypercompact AsCas12f1 nuclease showed markedly lower cytotoxicity than SpCas9 and enabled 100% gene deletion efficiency with preferred PAMs (TTTG, CTTG, GTTG). Second, we strengthened the shikimate pathway via multiple genetic strategies: overexpressing feedback-resistant DAHP synthase (*aroG*^*fbr*^) and chorismate mutase/prephenate dehydrogenase (*tyrA*^*fbr*^), as well as knocking out *pheA*. This manipulation blocks the phenylalanine synthetic branch and redirects metabolic flux toward the l-tyrosine branch. Third, we engineered the branched-chain fatty acid (BCFA) pathway via promoter replacement of *bkdA2B2C2*, *LipAB*, *fabF* and deletion of *acdH* to enhance isododecanoyl side-chain supply. The combinatorial engineering yielded strain B-13, which produced 1740 mg/L A40926B0 in shake flasks. Finally, 50-L fed-batch fermentation with continuous maltodextrin feeding further increased the titer to 1817 mg/L, the highest reported titer to date. This work establishes a robust CRISPR editing tool for *N. gerenzanensis* and provides valuable engineering references for precursor-oriented strain improvement targeting lipoglycopeptide antibiotics, offering insights for the industrial scale production of A40926B0.

## Graphical abstract

**Figure:**
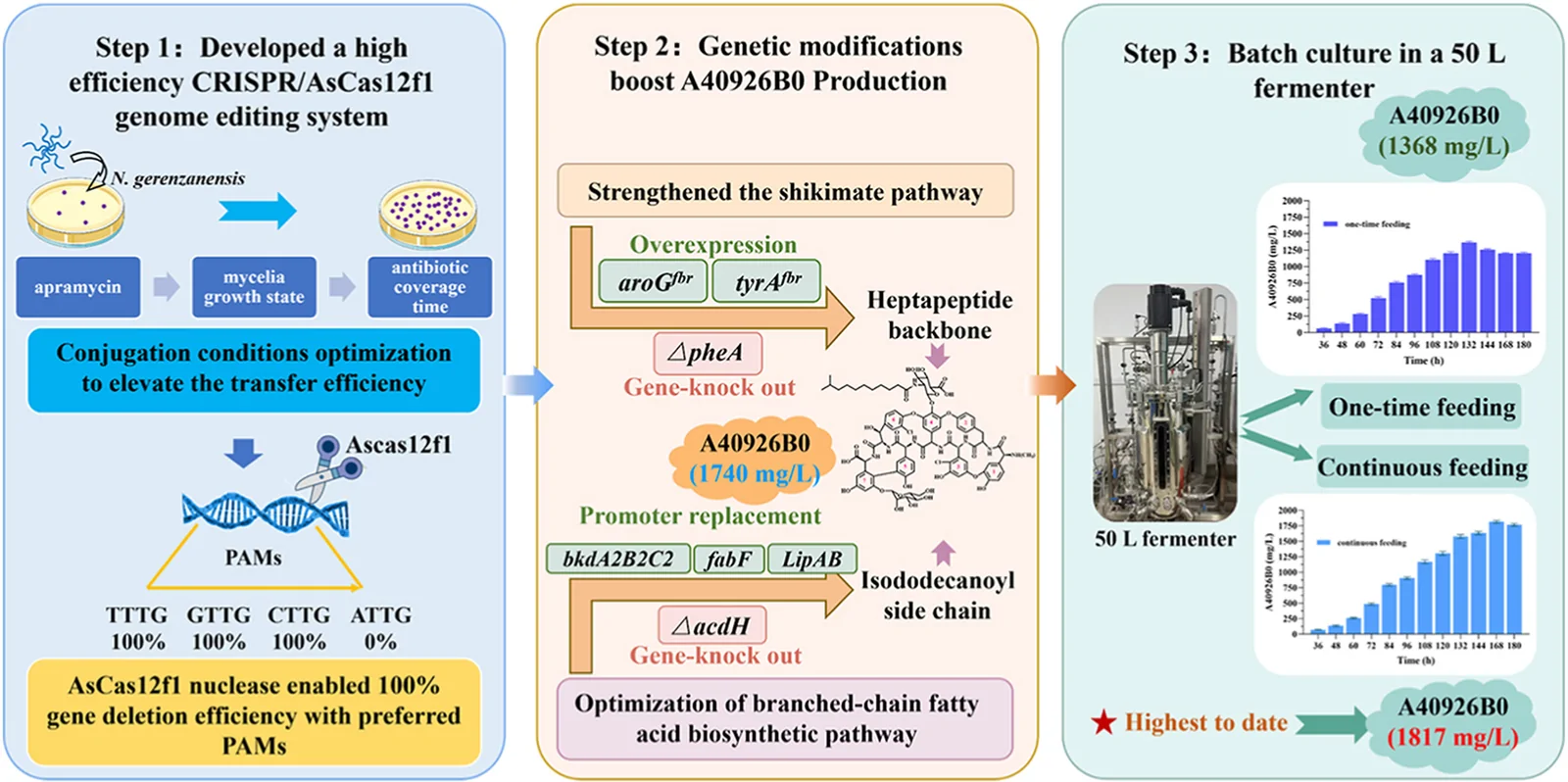

## 1 Introduction

Rare actinomycetes, representing non-streptomycete actinobacteria, produce numerous secondary metabolites with diverse chemical scaffolds and remarkable bioactivities [1]. Nevertheless, target bioactive metabolite titers from most rare actinomycetes remain insufficient for large-scale industrial manufacturing. Accordingly, the development of an efficient genome-editing system is an essential prerequisite for achieving high-level overproduction of secondary metabolites in these biotechnologically valuable microbes. Compared with model streptomycetes such as *Streptomyces coelicolor*, most rare actinomycetes are recalcitrant to genetic manipulation [2]. Major barriers include low intergeneric conjugation efficiency, severe cytotoxicity of conventional CRISPR-Cas systems that limits their practical application, and labor-intensive screening for double-crossover recombinants. These obstacles make multiplex genetic modification particularly challenging for this group, thereby severely constraining strain improvement and the dissection of biosynthetic mechanisms. New genome-editing tools for rare actinomycetes are therefore highly desirable to advance strain engineering toward the biosynthesis of industrially valuable natural products.

In recent years, a series of hypercompact type V-F CRISPR/Cas12f systems have been extensively identified, whose nucleases range from 400 to 700 amino acids (aa) and possess intrinsic target-specific double-stranded DNA (dsDNA) cleavage activity [3]. Relative to other reported Cas effectors, Cas12f nucleases exhibit prominent advantages for genomic engineering, which stem primarily from their compact molecular size and excellent intracellular deliverability [[3], [4], [5]]. Among these Cas12f orthologs, AsCas12f1, a 422-aa nuclease derived from *Acidibacillus sulfuroxidans*, is one of the most well-characterized homologs. In vitro biochemical assays have verified that AsCas12f1 achieves its optimal dsDNA cleavage efficiency at 45–55°C, while its catalytic activity is markedly attenuated under low-temperature conditions [3,6]. This temperature-dependent enzymatic characteristic indicates that AsCas12f1 can act as a low-cytotoxicity nuclease for mesophilic actinomycetes whose optimal growth temperature is around 30°C. Beyond its fundamental functional characterization, this compact system has been successfully engineered into potent genome editing platforms, with validated applicability across diverse eukaryotic cells including plant and human cells [3,7], and several prokaryotic strains, such as *Escherichia coli*, *Bacillus anthracis* and *Streptomyces coelicolor* [4,5,8]. Despite these successful implementations, the editing performance of AsCas12f1 has not been evaluated in rare actinomycetes.

As a novel generation antibiotic, dalbavancin is clinically utilized to treat infections induced by multidrug-resistant Gram-positive pathogens, particularly those induced by vancomycin-resistant bacterial strains [[9], [10], [11]]. Lipoglycopeptide A40926, the critical precursor of the semi-synthetic antibiotic dalbavancin, is a complex of structurally analogous compounds that differ solely in their linked hydrophobic acyl side chains [12,13]. In accordance with the approval requirements for dalbavancin established by the U.S. Food and Drug Administration (FDA), component B0 featuring an isododecanoyl side chain must account for more than 75% of the total complex [[14], [15], [16]]. The rare actinomycete *Nonomuraea gerenzanensis* ATCC 39727 is the only characterized natural microbial host carrying the complete *dbv* (from dalbavancin) biosynthetic gene cluster, which comprises 37 open reading frames (ORFs) dedicated to A40926B0 biosynthesis. This lipoglycopeptide is assembled via a nonribosomal peptide synthetase (NRPS) machinery that incorporates seven amino acid monomers, two sugar moieties (mannose and glucuronic acid residues), and an isododecanoyl fatty acid side chain to construct the full A40926B0 scaffold [17]. To date, fermentation is the only industrial route to obtain A40926B0 due to its intricate chemical structure. Nevertheless, wild-type strains yield low A40926B0 titers stemming from three major bottlenecks: inadequate NRPS expression, impaired function of regulatory genes, and restricted biosynthesis of amino acid and fatty acid precursors; these limitations collectively impede the industrial-scale production and commercialization of A40926B0 and its derivatives [18]. Previous studies have improved A40926B0 titers or the purity of the B0 component through multiple strategies: overexpressing core *dbv* biosynthetic genes, blocking acetylation side reactions, duplicating the *dbv* cluster, rewiring regulatory networks, and optimizing fermentation parameters [[18], [19], [20]]. These strategies focus exclusively on structural and regulatory genes within the biosynthetic cluster, while upstream precursor-supply-related genes for amino-acid and fatty-acid formation remain largely uninvestigated in *N. gerenzanensis*. Fatty acid chains determine lipopeptide pharmacokinetic properties and antibacterial potency, and multiple studies on other lipopeptides have validated that strengthening fatty acid synthesis pathways effectively elevates product titers [[21], [22], [23], [24]]. Meanwhile, intracellular amino acid pools directly limit NRPS catalytic flux, and reinforcing amino acid biosynthetic routes has also proven efficient for boosting lipopeptide yields in reported systems [22,25]. Further investigation of these upstream metabolic routes may offer new opportunities for strain engineering in *N. gerenzanensis*.

The routine genetic manipulation in *N. gerenzanensis* ATCC 39727 relies on intergeneric conjugation mediated by the site-specific integrative plasmid pSET152 [26]. However, the conjugation efficiency remains unsatisfactorily low, and gene knockout requires screening for double-crossover mutants, making large-scale multi-gene genetic modification impractical in this strain [19]. Furthermore, the mainstream CRISPR/Cas9 gene editing system is almost ineffective in *N. gerenzanensis* ATCC 39727, thus creating an urgent demand for efficient gene editing tools. Given its hypercompact size, potentially low cytotoxicity, and broad applicability in diverse prokaryotic and eukaryotic cells, the CRISPR/AsCas12f1 system represents an attractive candidate for constructing efficient genome-editing platforms for *N. gerenzanensis*, and provides valuable insights into genetic modification in this underexplored microbial group.

In this study, we optimized the precursor supply involved in A40926B0 biosynthesis by leveraging the CRISPR/AsCas12f1 gene editing system. First, we comprehensively optimized the conjugation transfer process of *N. gerenzanensis* ATCC to improve conjugation efficiency. Second, an efficient CRISPR/AsCas12f1-based gene editing system was successfully applied in *N. gerenzanensis* for the first time. Third, we remodeled the shikimate pathway to boost aromatic precursor pools, including the overexpression of feedback-resistant variants of DAHP synthase and chorismate mutase/prephenate dehydrogenase variants *tyrA*^*fbr*^ alongside *pheA* deletion to synergistically improve A40926B0 yields. Fourth, we tuned the branched-chain fatty acid (BCFA) biosynthetic pathway which generates the isododecanoyl side chain of A40926B0, by promoter engineering of key lipid genes and *acdH* knockout to further raise A40926B0 titers. Finally, the engineered strain B-13 was cultured in a 50 L fermenter with optimized maltodextrin fed-batch feeding to stabilize pH, achieving the highest A40926B0 titer of 1817 mg/L to date and demonstrating its industrial application potential.

## 2 Materials and methods

### 2.1 Strains, plasmids and culture conditions

The strains and plasmids used in this study are listed in Table S1 and primers in Table S2. *Escherichia coli* DH5α and *E. coli* ET12567/pUZ8002 were employed as the host strain for plasmid construction and the donor strain for intergeneric conjugation, respectively. *E. coli* strains were grown at 37°C in Luria-Bertani (LB) broth or on LB agar plates. N. gerenzanensis D11, a mutant strain of *N. gerenzanensis* ATCC 39727, was stored in our laboratory and used as the parental strain in this study. *N. gerenzanensis* D11 and its derivatives were grown at 29°C. For intergeneric conjugation, the mycelia of *N. gerenzanensis* were obtained from four different liquid media, including VSP [27], YEME [28], R3 [29] and tryptone soya broth (TSB). MS solid medium [28] was used to observe the spore formation of interspecies conjugation. When necessary, antibiotics were added at the following concentrations: 50 μg/mL apramycin, 25 μg/mL chloramphenicol, 25 μg/mL kanamycin, and 50 μg/mL nalidixic acid. For fermentation, *N. gerenzanensis* D11 and its derivatives were cultured on YMG solid medium [30] and grown for 7 d. Then, the mycelium was inoculated in seed medium (3% maltodextrin, 1% soybean powder, 1% yeast extract, 0.1% MgSO_4_·7H_2_O) and grown on a rotary shaker (220 rpm) for 3 d. A total of 3 mL (10% v/v) of seed culture was transferred into flasks containing 30 mL of fermentation medium (6% maltodextrin, 2% soybean powder, 1% yeast extract, 0.1% MgSO_4_·7H_2_O, 0.2% NaCl, 0.01% CuSO_4_, 0.01% FeSO_4_, 0.2% CaCO_3_) and then cultured on a rotary shaker (220 rpm) for 7 d.

### 2.2 Fed-batch fermentation in a 50-L fermenter

Fed-batch fermentation was performed in a 50-L fermenter containing 30 L of fermentation medium, which was prepared as described above and was consistent with the formulation used in shake flask cultures. *N. gerenzanensis* was first cultured in 150 mL of seed medium in 1-L shake flasks at 29°C with shaking at 220 rpm for 48 h. Subsequently, 300 mL of the seed culture (5% inoculum) was transferred to a 10-L seed fermenter containing 6 L of seed medium. Following this, 3 L of the secondary seed culture (10% inoculum) was inoculated into the aforementioned 50-L bioreactor with 30 L of fermentation medium. Throughout the entire fermentation process, the aeration rate was set at a constant 0.5 vvm (air volume per culture volume per minute), and the dissolved oxygen (DO) level was maintained at no less than 30%. During the fermentation process, two feeding strategies for maltodextrin—one-time feeding and continuous feeding—were adopted to alleviate carbon source deficiency and improve A40926B0 yield.

### 2.3 Plasmid construction and conjugation

To investigate the length of the homologous arms, different lengths of downstream/upstream homologous arms of biosynthesis gene *dbv23* were amplified using primer pairs *dbv23*-U-F1/R1 and *dbv23*-D-F1/R1 for 1.0 kb, *dbv23*-F-F2/R1 and *dbv23*-D-F1/R2 for 1.5 kb, *dbv23*-F-F3/R1 and *dbv23*-D-F1/R3 for 2.0 kb, *dbv23*-F-F4/R1 and *dbv23*-D-F1/R4 for 2.5 kb, respectively. Then, these homologous arms were inserted into the *Xba*I-*Bam*HI site of suicide plasmid pSET153 [31].

The CRISPR genome editing system in *N. gerenzanensis* was constructed based on the temperature-sensitive plasmid pKC1139. The *AsCas12f1* gene originating from *Acidibacillus sulfuroxidans* was codon optimized and synthesized. With pAsCas12f1-T plasmid serving as the PCR template, a DNA fragment harboring the *tipA*-driven AsCas12f1 gene and the *PJ23119*-promoted transcription cassette of a synthetic sgRNA scaffold (sgRNA_V1) was amplified by PCR using the Cas12-F/D primer pair. The PCR product was cloned into pKC1139 between *Hin*dIII and the *Eco*RI site to yield pCas12f1^−*tipA*^. Subsequently, the *gapdh* and ermE*p promoters were separately amplified by PCR using the primer pairs *gapdh*-F/R and ermE*p-F/R, with plasmids p*gapdh*-T and permE*p-T serving as the respective templates. The resulting PCR products were then cloned into the *Xba*I and the *Nde*I restriction sites of pCas12f1^−*tipA*^, yielding pCas12f1^−*gapdh*^ and pCas12f1^−ermE^*^p^. The pCas12f1-based editing plasmids containing the sgRNA transcription cassettes and homologous arms (HAs) were constructed as follows. Here, the construction of the editing plasmid with *dbv4*-targeting sgRNA (*sg1*) for *dbv4* deletion is given as an example. The *dbv4* gene-specific sgRNA was amplified from pCas12f1^−*tipA*^ with the primers *dbv4*sgRNAF/R. The PCR product was cloned into pCas12f1^−*tipA*^ between *Hin*dIII and the *Spe*I site to yield pCas12f1^−*gapdh*^-*sg*^*dbv4*^. Two homologous arms flanking the *dbv4* ORF were amplified from *N. gerenzanensis* genomic DNA separately with the primer pairs, *dbv4*LF/*dbv4*LR, and *dbv4*RF/*dbv4*RR. The resulting PCR products were ligated to the *Hin*dIII linearized pCas12f1^−*gapdh*^-*sg*^*dbv4*^ using the Exnase II One Step Cloning Kit (Vazyme Biotech, Nanjing, China), resulting in pCas12f1^−*gapdh*^-*sg*^*dbv4*^-HA. The SpCas9-based editing plasmids harboring both sgRNA transcription cassettes and HAs, and control plasmids harboring only the sgRNA transcription cassettes, were constructed as reported previously [32]. Additionally, the construction process for integrated or overexpressed pCas12f1-donor plasmids was similar to that of knockout plasmid, with the target gene or gene expression cassette being assembled between the upstream and downstream homologous arms, respectively.

All the gene-editing plasmids were introduced into *N. gerenzanensis* by conjugation and were confirmed on selective plates. Subsequently, the exconjugants with the correct deletion of target genes were confirmed by colony PCR using the verification primer pairs. Additionally, the CRISPR system plasmids, which harbored the temperature-sensitive replicon pSG5, could be eliminated when the actinomycete was cultured at high temperature (37−39°C), facilitating the genome editing process.

### 2.4 Analysis

Fehling's reagent was used to measure total sugar concentrations. Biomass was recorded as packed mycelium volume (PMV): 10 mL fermentation broth was centrifuged at 3000 *g* for 15 min to measure compact mycelial volume.

The A40926B0 analysis referred to the method described by Huijun Dong [18] with modifications. Briefly, the fermentation broth sample was adjusted to pH11 by adding NaOH solution, and then sonicated at 50°C for 0.5 h. Subsequently, the sample was centrifuged at 3000 g for 5 min. The obtained supernatant was filtered through a 0.22 μm Millipore filter and analyzed by high-performance liquid chromatography (HPLC) on an SHIMAZU Shim-pack GIST C18 column (4.6 × 150 mm; 5 μm) and the analytic method was followed with acetonitrile/20 mM ammonium acetate solution (30:70, v/v) at a flow rate of 1 mL/min and UV detector at 210 nm using Shimadzu LC-20AT.

### 2.5 Quantitative RT-PCR

Harvested mycelia of *N. gerenzanensis* were subjected to two washes with RNase-free TE buffer prior to total RNA isolation. Total RNA was isolated via the Bacteria RNA Extraction Kit (Vazyme), strictly following the manufacturer-recommended protocols. Genomic DNA contamination was eliminated during reverse-transcription using the PrimeScript™ FAST RT reagent Kit with gDNA Eraser (Takara). Validated RNA templates were processed to generate first-strand cDNA according to supplier protocols. Subsequent qRT-PCR assays were carried out on a qTOWER^3^ G Real-Time PCR System (Analytik Jena GmbH, Jena, Germany) employing TB Green™ Premix Ex Taq™ II (TaKaRa).

### 2.6 Statistical analysis

Group differences in the number of exconjugants detected, as well as disparities in editing efficiency between SpCas9-and AsCas12f1-based tools, were analyzed via one-way analysis of variance (ANOVA) followed by Tukey's multiple comparison test. In all instances, differences with P < 0.05 were considered statistically significant. All experimental data were processed and analyzed using GraphPad Prism (Version 10.0) and Origin 2021 software.

## 3 Results and discussion

### 3.1 Optimization of the conjugation transfer conditions in N. gerenzanensis

**Fig. 1:**
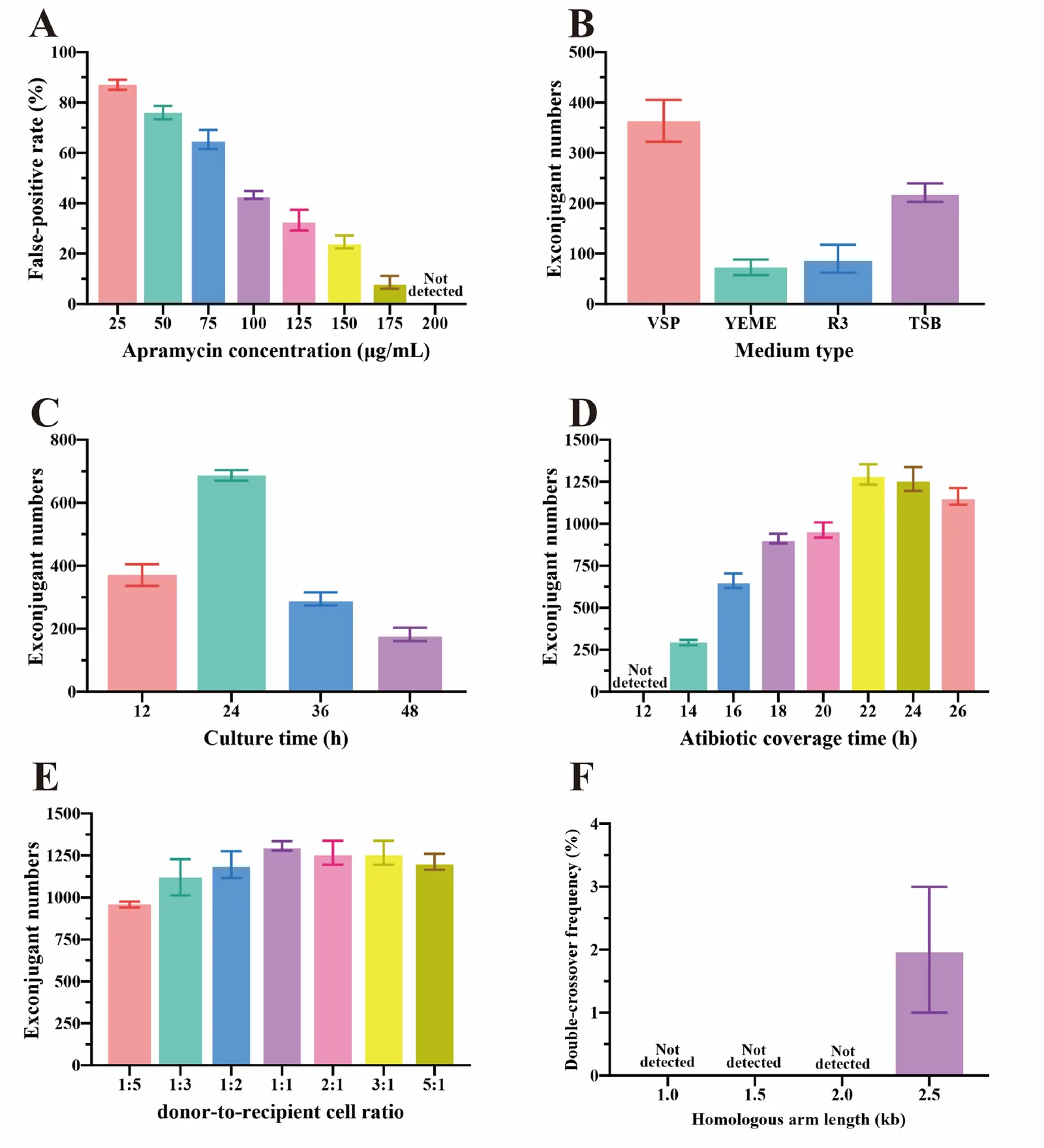
Optimization of conjugation transfer conditions in *N. gerenzanensis* D11. (A) Effect of apramycin concentration on the inhibition of false-positive conjugants. (B) Effects of different media (VSP, YEME, R3, TSB) on the number of exconjugants. (C) Effect of mycelial culture time on conjugation efficiency. (D) Effect of antibiotic coverage time on conjugation efficiency. (E) Effect of donor-to-recipient ratio on conjugation efficiency. (F) Effect of homologous arm length on gene knockout efficiency via homologous recombination. Data are presented as mean ± SD (standard deviation) (n = 3).

The thorough study of *N. gerenzanensis* at the molecular level is limited by its inefficient genetic manipulation system. Protoplast transformation has been used for the gene transfer of *N. gerenzanensis*, but the preparation, regeneration, and transformation of protoplast are tedious [27]. Another routine genetic manipulation of *N. gerenzanensis* is intergeneric conjugation. However, the conjugation efficiency remained below 10^−8^, indicating multiple critical parameters—apramycin concentration, mycelial growth state, antibiotic incubation duration, and donor-to-recipient cell ratio—require systematic optimization. The effect of apramycin concentration on conjugation transfer was studied firstly. The results showed that the false-positive conjugants were inhibited completely when the apramycin concentration was at 200 μg/mL (Fig. 1A). Previous studies have demonstrated that medium composition and cultivation duration jointly govern the physiological status and hyphal micromorphology of actinomycetal mycelia, which in turn modulates intergeneric conjugation efficiency [2]. Therefore, we investigated the effect of mycelia state on the intergeneric conjugation of *N. gerenzanensis* in different media including VSP, YEME, R3 and TSB. As shown in Fig. 1B, the exconjugant counts of VSP were the highest (364 ± 41), while those of other media were less than 90. VSP medium delivered the highest conjugation efficiency, which could be attributed to its balanced nutrient profile that supports vigorous mycelial growth and maintains robust metabolic activity in *N. gerenzanensis*. Micromorphological imaging further revealed that strains cultured in VSP formed loose reticular mycelial networks that generated ample contact surfaces between donor and recipient cells to promote conjugation (Fig. S1A). While *N. gerenzanensis* grows readily in YEME, R3, and TSB media, these cultures tended to form dense mycelial pellets. Consistent with this, microscopic analysis demonstrated that the compact morphology observed in the three media provided only limited cell-cell contact, thereby hindering intergeneric conjugation (Fig. S1A). The optimal mycelial culture time was 24 h (Fig. 1C), which corresponds to the exponential growth phase of *N. gerenzanensis* (Fig. S1B) where mycelia are metabolically active and more competent for foreign DNA uptake. Culture times longer than 36 h led to mycelial senescence and reduced viability, while those shorter than 12 h resulted in insufficient biomass, both of which decreased conjugation efficiency. Furthermore, we optimized both the conjugation incubation duration and donor/recipient mixing ratio. The results showed that the optimal incubation period was approximately 22 h (Fig. 1D and E), whereas a 1:1 donor-to-recipient ratio delivered the maximum conjugation efficiency. Collectively, optimization of all tested experimental variables markedly improved intergeneric conjugation efficiency in *N. gerenzanensis*, laying a robust technical foundation for subsequent genome engineering of this strain. This refined conjugation protocol can serve as a versatile approach for genetic transformation of other rare actinomycetes susceptible to dense mycelial pellet formation.

Finally, the gene-editing efficiency of plasmid tools with different lengths of homologous arms based on the optimal intergeneric conjugation conditions was evaluated. As shown in Fig. 1F, detectable conjugants were only recovered when homologous arms extended to 2.5 kb for the editing of *dbv23*. We confirmed this trend by repeating the assay on a separate target, *dbv4*, which generated fully matching data. The process of obtaining gene knockouts through homologous double-crossover recombination by antibiotic-free culturing is laborious and time-consuming. In this process, the absence of selective pressure could cause conjugants to revert to the wild-type allele via homologous recombination. In this study, only six gene-knockout mutants were observed among 300 conjugants, indicating that the efficiency of gene knockout via homologous recombination was extremely low. Therefore, establishing a more efficient genetic manipulation method is essential.

### 3.2 Application of CRISPR/AsCas12f1-based genome editing in N. gerenzanensis

AsCas12f1 is a miniature Type V–F CRISPR/Cas effector active in a broad range of prokaryotic and eukaryotic hosts, with intrinsically low cytotoxicity against microorganisms. Taking these merits into account, we comprehensively characterized three key properties of this nuclease in *N. gerenzanensis* D11: cellular toxicity, PAM preference, and genome-editing performance. First, we evaluated the toxicity effects of AsCas12f1 on *N. gerenzanensis* D11 targeting the *dbv4* gene (encoding a positive regulatory protein for the biosynthesis of A40926, inactivation of dbv4 abolished A40926 biosynthesis) with SpCas9 included as a control. The codon-optimized AsCas12f1 gene was cloned into pKC1139 under the control of two strong constitutive promoters (*gapdh* and ermE*p) and one inducible promoter (*tipA*), yielding pCas12f1^−*gapdh*^, pCas12f1^−ermE^*^p,^ and pCas12f1^−*tipA*^. Based on the above three plasmids, the recombinant plasmids pCas12f1^−*gapdh*^-*sg*^*dbv4*^, pCas12f1^−ermE^*^p^-*sg*^*dbv4*^ and pCas12f1^−*tipA*^-*sg*^*dbv4*^ containing transcription cassettes of *sg* ^*dbv4*^ (driven by the strong constitutive J23119 promoter) were constructed. Meanwhile, parallel plasmids were generated for SpCas9, with the SpCas9 gene cloned into pKC1139 under the same three promoters to create pCas9^−*gapdh*^, pCas9^−ermE^*^p^ and pCas9^−*tipA*^, followed by introduction of the *J23119*-driven gene-specific sgRNA to produce the corresponding recombinant plasmids (pCas9^−*gapdh*^-*sg*^*dbv4*^, pCas9^−ermE^*^p^-*sg* ^*dbv4*^, pCas9^−*tipA*^-*sg* ^*dbv4*^). The above plasmids, along with the control plasmid pKC1139, were transferred into *N*. *gerenzanensis* D11 via intergenic conjugation. The efficiency of conjugal transfer was then compared between the SpCas9-based and AsCas12f1-based editing systems.

**Fig. 2:**
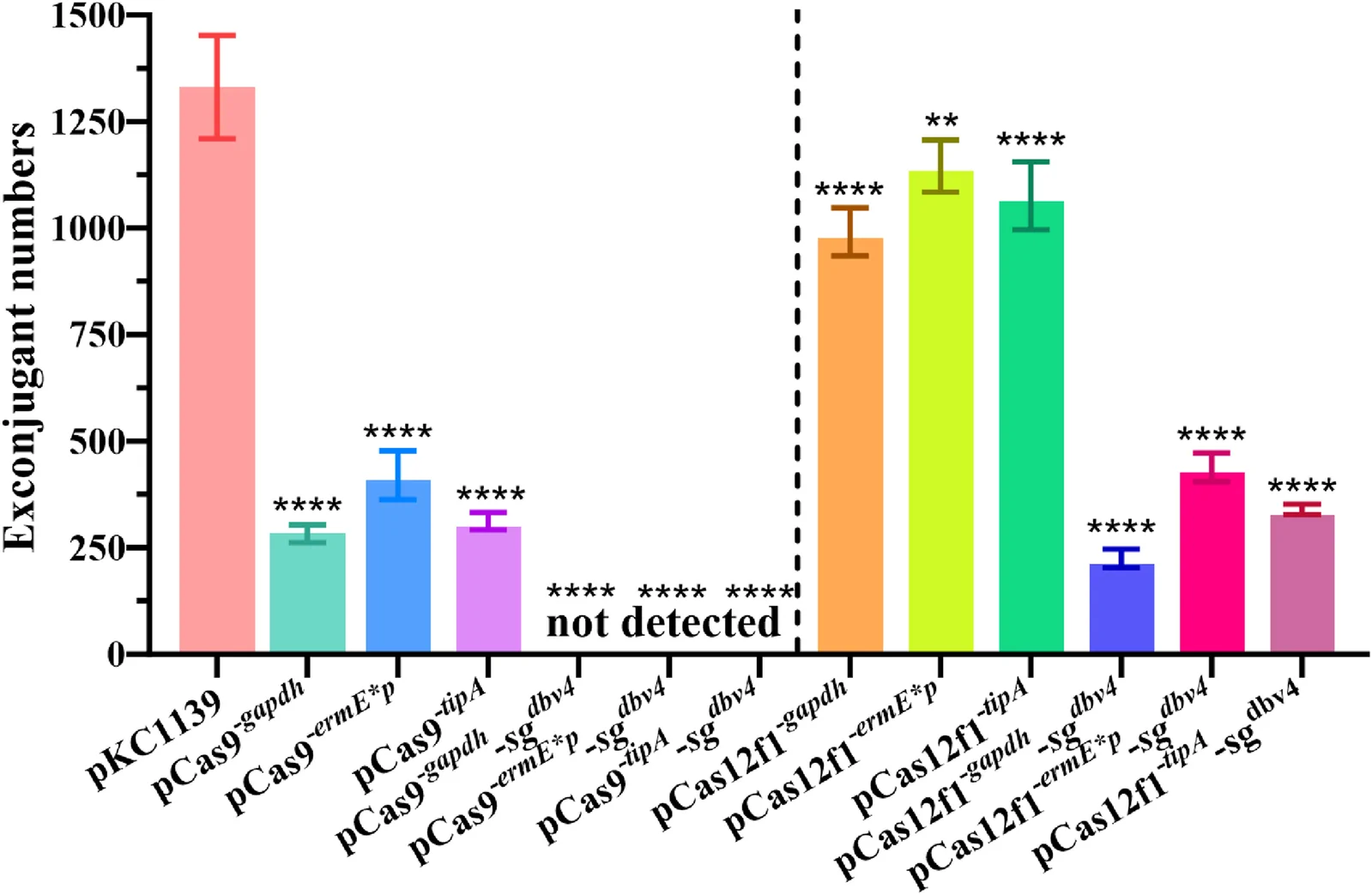
Comparison of cytotoxicity and conjugation efficiency between AsCas12f1 and SpCas9 systems in *N. gerenzanensis* D11. Relative numbers of exconjugants obtained with plasmids expressing AsCas12f1 or SpCas9 under different promoters (*gapdh*, ermE*p, tipA) with or without sgRNA targeting *dbv4*. The empty vector pKC1139 was used as control. In each experiment, 10^8^ *N. gerenzanensis* mycelium fragments were used. Data are mean ± SD (n = 3). **P < 0.01,****P < 0.0001 by one-way ANOVA + Tukey's multiple comparison test (control column: pKC1139).

As shown in Fig. 2, the exconjugant counts decreased significantly after the introduction of pCas9^−*gapdh*^, pCas9^−ermE^*^p^ and pCas9^−*tipA*^ compared with the empty vector pKC1139 (accounting for 21.3%, 31.6%, and 23.5% of that of pKC1139, respectively). However, the introduction of pCas12f1^−*gapdh*^, pCas12f1^−ermE^*^p^ and pCas12f1^−*tipA*^ only resulted in slightly reduced exconjugant counts (74.4%, 86.1% and 80.8% of that of pKC1139). The different number of exconjugants produced by the expression of SpCas9 and AsCas12f1 clearly demonstrated that AsCas12f1 exhibited lower toxicity than SpCas9 toward *N. gerenzanensis* D11. This reduced cytotoxicity of AsCas12f1 can be primarily attributed to its exceptionally compact protein size (only 422 amino acids), which imposes less metabolic burden on host cells and lowers unintended off-target cleavage compared with the large SpCas9 (1368 amino acids) [3]. Moreover, the weak intrinsic DNA cleavage activity of AsCas12f1 under routine culture conditions further alleviates toxic double-strand DNA breaks, making it more suitable for genetic manipulation in *N. gerenzanensis* D11 [33].

When introducing the plasmids pCas9^−*gapdh*^-*sg*^*dbv4*^, pCas9^−ermE^*^p^-*sg*^*dbv4*^, pCas9^−*tipA*^-*sg*^*dbv4*^ into D11, no exconjugants were obtained (Fig. 2). By contrast, the control strain bearing a SpCas9-only plasmid devoid of the sgRNA scaffold produced visible exconjugants but exhibited drastically lower exconjugant counts than the empty-vector control (Fig. 2), confirming that free SpCas9 protein imposes marked intrinsic cytotoxicity of free SpCas9. Nevertheless, this cytotoxicity alone fails to account for complete growth failure. The lethal phenotype mainly stems from target specific genomic double strand breaks (DSBs) introduced by the SpCas9 sgRNA ribonucleoprotein complex, instead of nonspecific DNA interactions or off target effects from free SpCas9 protein [34]. Unlike SpCas9 systems, transformation with three AsCas12f1-sgRNA plasmids (pCas12f1^−*gapdh*^-*sg*^*dbv4*^, pCas12f1^−ermE^*^p^-*sg* ^*dbv4*^ and pCas12f1^−*tipA*^-*sg* ^*dbv4*^) yielded viable exconjugants, with average colony numbers of 225 ± 22, 438 ± 34 and 340 ± 12, respectively (Fig. 2). As a thermophilic miniature CRISPR nuclease, AsCas12f1 exhibits drastically reduced DNA cleavage activity at 29°C, far below its optimal temperature range (45–55°C), thus minimizing toxic DSBs and enabling host cell survival [3]. In addition, ermE*p-driven expression of AsCas12f1 exhibited lower host toxicity than that mediated by *gapdh* and *tipA* in the presence of the gene-specific targeting sgRNA, indicating that the moderate and stable expression strength of the ermE*p promoter effectively avoids overexpression-related toxicity and is therefore more suitable for subsequent genome editing applications in this strain.

**Fig. 3:**
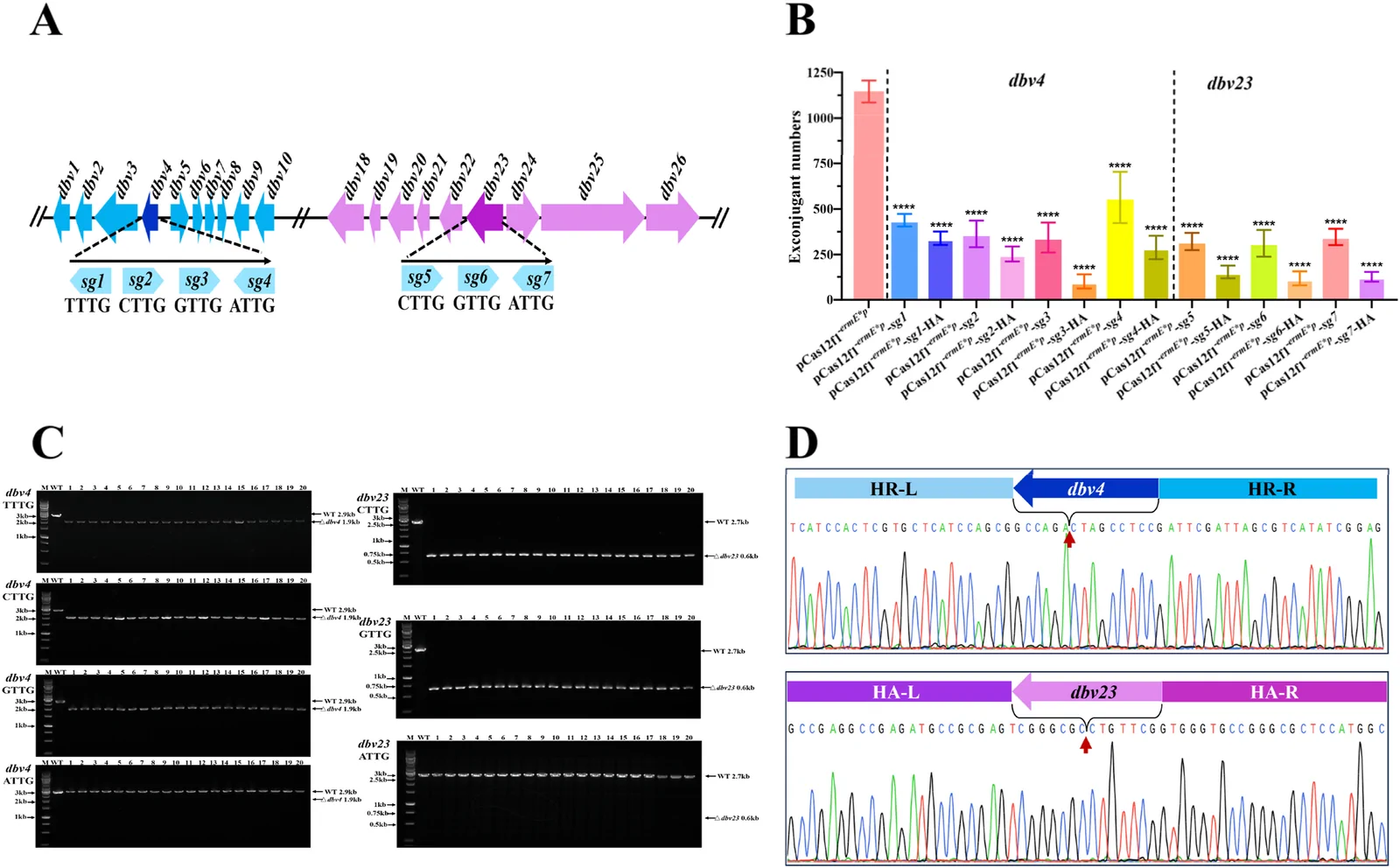
Construction and evaluation of the CRISPR/AsCas12f1-mediated gene editing system in *N. gerenzanensis* D11. (A) Schematic of sgRNA target sites and corresponding PAM sequences for *dbv4* and *dbv23* deletion. (B) Conjugal transfer efficiency of AsCas12f1-based editing plasmids using sgRNAs with different PAMs. In each conjugal transfer experiment, 10^8^ *N. gerenzanensis* mycelium fragments were used. Data are mean ± SD (n = 3). ****P < 0.0001 by one-way ANOVA + Tukey's multiple comparison test (control column: pCas12f1^−ermE^*^p^). (C) Colony PCR analysis of gene deletion efficiency mediated by the AsCas12f1 system with different PAMs. (D) DNA sequencing verification of *dbv4* and *dbv23* deletion mutants.

Next, to test the gene-editing efficiency of AsCas12f1 in *N. gerenzanensis*, two genes including *dbv4* and *dbv23* (encoding an acetyltransferase involved in the biosynthesis of A40926, its deletion would induce an increase in A40926 production) were selected. The 5′-NTTG protospacer adjacent motifs (PAMs) were found to be essential for AsCas12f1-mediated DNA cleavage, and the -N nucleotide at this site exerted a significant impact on targeting activity, following the order of T > C > A > G [4]. However, T-rich PAMs exhibit a relatively low distribution frequency in actinomycetes harboring high GC-content genomes. The 5′-NTTG PAM motifs were analyzed in the genome of *N. gerenzanensis* L70 (12,010,032 bp; GenBank accession: CP084058.1), which encodes 11,220 genes with an average length of 1070 bp. Statistical quantification showed an average of 0.6-TTTG, 0.6-ATTG, 5.2-CTTG and 5.1-GTTG sites per gene. When normalized to per 1000 bp of genomic sequence, the average counts were 0.5-TTTG, 0.5-ATTG, 4.9-CTTG and 4.8-GTTG sites, respectively. The low abundance of 5′-TTTG and 5′-ATTG greatly limits the broad application of AsCas12f1 in this strain. Given this uneven PAM distribution, we tested all four 5′-NTTG variants to expand available targeting sites and comprehensively characterize their editing performance. Therefore, in addition to TTTG, three other PAMs (CTTG, ATTG, and GTTG) were also employed for gene disruption. For the deletion of *dbv4*, four sgRNAs were designed to target distinct positions, each associated with a specific PAM (TTTG, CTTG, GTTG, or ATTG) (Fig. 3A). Using these constructs above as backbones, two sets of recombinant plasmids were generated: (i) pCas12f1^−ermE^*^p^-*sg1* (*sg1* is the same as *sg* ^*dbv4*^ described above), *pCas12f1*^−ermE^*^p^*-sg2*, pCas12f1^−ermE^*^p^-*sg3* and pCas12f1^−ermE^*^p^-*sg4* which harbor gene-specific sgRNAs under the control of the J23119 promoter; and (ii) pCas12f1^−ermE^*^p^-*sg1*-HA, pCas12f1^−ermE^*^p^-*sg2*-HA, pCas12f1^−ermE^*^p^-*sg3*-HA and pCas12f1^−ermE^*^p^-*sg4*-HA, which additionally contain two homologous arms (HAs). For the deletion of *dbv23* (which lacks the TTTG motif), three sgRNAs were designed to target distinct positions, each associated with a specific PAM (CTTG, GTTG, or ATTG) (Fig. 3A). Relating recombinant plasmids (pCas12f1^−ermE^*^p^-*sg5*, pCas12f1^−ermE^*^p^-*sg6*, pCas12f1^−ermE^*^p^-*sg7*, pCas12f1^−ermE^*^p^-*sg5*-HA, pCas12f1^−ermE^*^p^-*sg6*-HA and pCas12f1^−ermE^*^p^-*sg7*-HA) were generated. Totally, seven editing plasmids pCas12f1^−ermE^*^p^-*sg*1-7/HA and seven corresponding recombinant plasmids without HAs (pCas12f1^−ermE^*^p^-*sg*1-7) were constructed. Then, the efficiency of conjugal transfer and gene editing was determined.

The introduction of these recombinant editing plasmids produced variable exconjugant counts, most of which were markedly lower than those obtained with the control plasmid pCas12f1^−ermE^*^p^ (Fig. 3B). Of all constructs, pCas12f1^−ermE^*^p^-*sg*3/HA (GTTG) and pCas12f1^−ermE^*^p^-sg6/HA (GTTG) exhibited the lowest exconjugant yields. This reduction in exconjugant numbers can be attributed to growth retardation triggered by sgRNA-guided AsCas12f1 cleavage and the subsequent homologous recombination repair. To evaluate gene deletion efficiency, 20 exconjugants were randomly selected from each conjugation plate. Correspondingly, PCR analysis validated that the AsCas12f1-mediated editing system attained a deletion efficiency of 100% by using TTTG, CTTG and GTTG PAMs (*sg1-sg3, sg5-sg6*) (Fig. 3C). No editing events were detected for sgRNA with PAM ATTG (*sg4*, *sg7*). This phenomenon was inconsistent with the results observed in *Escherichia coli*, indicating that rare-actinomycetes *N. gerenzanensis* with high GC content (72%) possesses different preferences for PAM. Owing to the plentiful CTTG and GTTG within the *N. gerenzanensis* genome, we confirmed that the AsCas12f1-driven editing enables highly efficient genome editing in this rare actinomycete. DNA sequencing of the PCR products and fermentation product analysis of the recombinant strains further confirmed the correct deletion of *dbv4* or *dbv23* (Fig. 3D, Fig. S2). Additionally, deletion of *dbv23* in *N. gerenzanensis* significantly increased the production of A40926B0, from 200 mg/L in the parental strain D11 to 400 mg/L in the derived mutant strain B-1. As reported previously, Dbv23 is an acetyltransferase that catalyzes O-acetylation of the mannose moiety in A40926B0 [35]. Given that the acetylated product exerts potent feedback inhibition on its own biosynthetic pathway, the Δ*dbv23* mutant achieves a twofold increase in product titer by relieving this negative regulatory effect. Therefore, the *N. gerenzanensis* B-1 was used as a chassis strain in the following experiments.

Finally, we assessed the mismatch tolerance of AsCas12f1-mediated genome editing by randomly introducing single- or double-nucleotide mismatches into the sgRNA guide sequence (Fig. S3). After conjugation and screening, no strains with target-knockout genotypes were obtained when 1–2-nt mismatches existed in sgRNA. These findings demonstrated that AsCas12f1 exhibits high editing fidelity in this high-GC rare actinomycete, as merely 1–2-nt mismatches can fully inactivate this nuclease and markedly lower the risks of off-target cleavage.

### 3.3 Enhancement of the shikimate pathway to promote A40926B0 synthesis

**Fig. 4:**
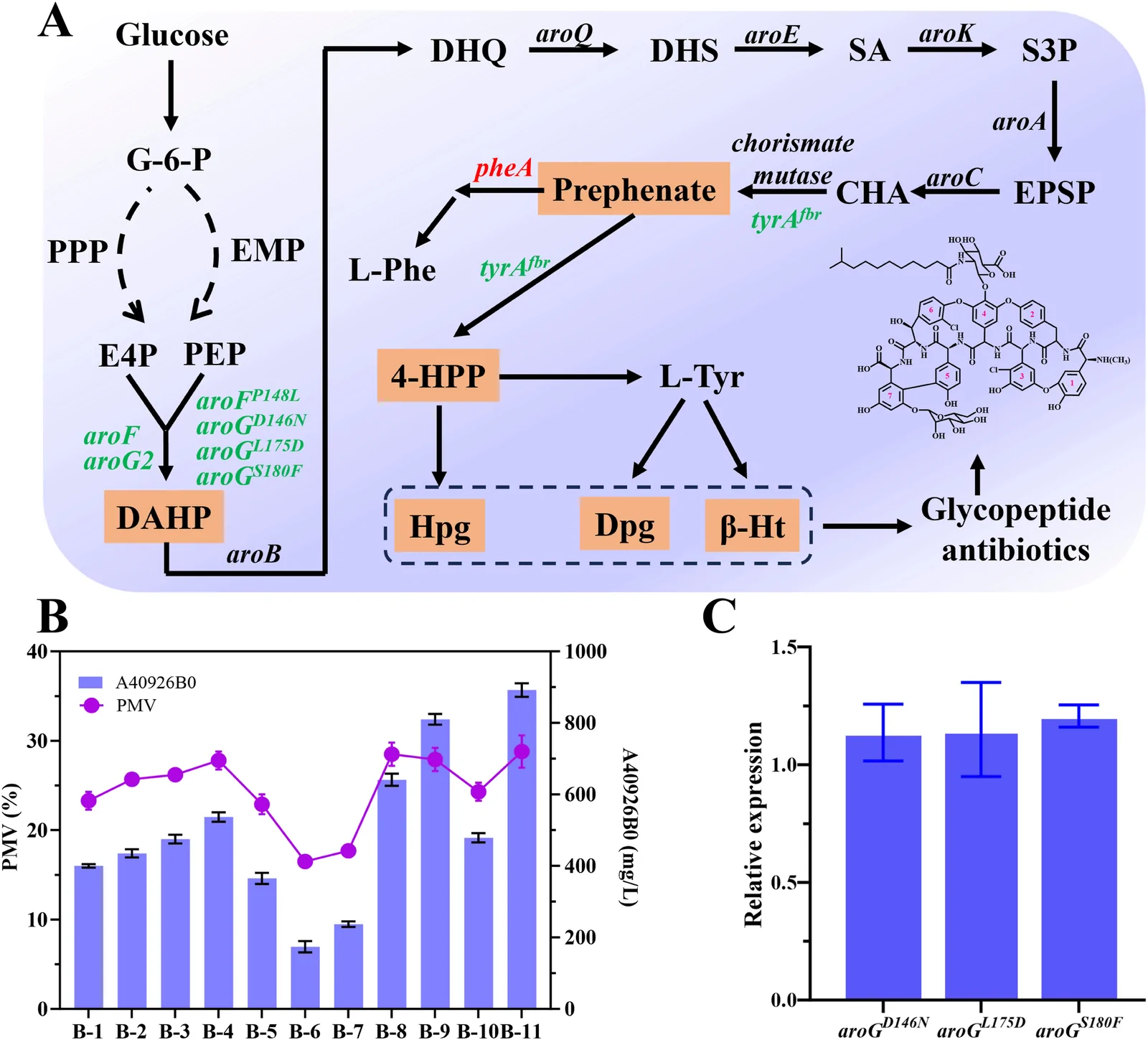
Engineering of the shikimate pathway to enhance A40926B0 biosynthesis in *N. gerenzanensis*. (A) Schematic overview of the shikimate pathway and aromatic amino acid precursor supply for A40926B0 biosynthesis. Glucose is catabolized via the glycolytic (EMP) and pentose phosphate (PPP) pathways to yield phosphoenolpyruvate (PEP) and erythrose 4-phosphate (E4P), which are condensed to 3-deoxy-d-arabino-heptulosonate-7-phosphate (DAHP) by DAHP synthase (encoded by *aroG/aroF*). Key enzymes include: *aroB* (dehydroquinate synthase), *aroQ* (dehydroquinate dehydratase), *aroE* (shikimate dehydrogenase), *aroK* (shikimate kinase), *aroA* (5-enolpyruvylshikimate-3-phosphate synthase), and *aroC* (chorismate synthase), which sequentially convert DAHP to chorismate (CHA). Chorismate is converted to prephenate by chorismate mutase (encoded by *tyrA*). Prephenate is metabolized to l-phenylalanine (L-Phe) by prephenate dehydratase (encoded by *pheA*), or to 4-hydroxyphenylpyruvate (4-HPP) by prephenate dehydrogenase (encoded by *tyrA*), which is further converted to l-tyrosine (L-Tyr). L-Tyr and its derivatives (Hpg, Dpg, β-Ht) serve as key precursors for the biosynthesis of the glycopeptide antibiotic A40926B0. Feedback-resistant variants *aroG*^*fbr*^ (including *aroG*^*D146N*^, *aroG*^*L175D*^, *aroG*^*S180F*^) and *aroF*^*P148L*^ were used to relieve feedback inhibition of DAHP synthase. Feedback-resistant variants *tyrA*^*fbr*^ was used to relieve feedback inhibition of chorismate mutase synthase. (B) A40926B0 titers (blue bars, right y-axis) and packed mycelial volume (PMV, purple circles, left y-axis) of strains engineered with shikimate pathway modifications (B-1 to B-11). Data are presented as mean ± SD (n = 3). (C) The relative expression of *aroG*^*D146N*^, *aroG*^*L175D*^ and *aroG*^*S180F*^ in B-3, B-4 and B-5 respectively by qRT-PCR. The RNA samples were collected from cultures at 72 h in fermentation medium. The transcription level of *hrdB* was assayed as an internal control. Data are presented as mean ± SD (n = 3). No significant differences were observed among groups (ns, Tukey's multiple comparison test).

A40926B0 is a heptapeptide assembled via NRPS. Its peptide backbone consists of one proteinogenic amino acid l-tyrosine (L-Tyr) at position 2 and six non-proteinogenic amino acids: 4-hydroxyphenylglycine (Hpg) at positions 1, 4, and 5; 3,5-dihydroxyphenylglycine (Dpg) at positions 3 and 7; and β-hydroxytyrosine (β-Ht) at position 6 [17]. All seven aromatic amino acids are derived from primary metabolites through the shikimate pathway (Fig. 4A). Therefore, we hypothesized that the biosynthesis of A40926B0 was limited by the supply of precursors (4-HPP, 4-hydroxyphenylpyruvic acid, and L-Tyr) derived from the shikimate pathway. As the committed first enzyme of the shikimate pathway, 3-deoxy-D-arabino-heptulosonate 7-phosphate (DAHP) synthase is tightly subject to feedback inhibition by the aromatic amino acid L-Tyr and l-phenylalanine (L-Phe), and thereby functions as a major regulator of metabolic flux through the pathway [36]. Therefore, for boosting the metabolic flux through the shikimate pathway, the feedback-resistant variants of DAHP synthase were overexpressed in *N. gerenzanensis* B-1, respectively. For this we selected four variants of DAHP synthase, a mutated feedback-resistant AroF^P148L^ from *Escherichia coli* MK201 [37] and three mutant variants of AroG (AroG^D146N^, AroG^L175D^ and AroG^S180F^), also from *E. coli* [[38], [39], [40]]. The four heterologous DAHP synthases were all codon-optimized for efficient overexpression in *N. gerenzanensis* B-1, with endogenous DAHP synthases AroG2 and AroF included as controls. All the strains overexpressing DAHP synthase were evaluated for their ability to produce A40926B0. As shown in Fig. 4, A40926B0 production increased to 435 mg/L, 475 mg/L and 537 mg/L in recombinant strains B-2 (*aroF*^*P148L*^), B-3 (*aroG*^*D146N*^) and B-4 (*aroG*^*L175D*^), respectively. While A40926B0 production decreased to 365 mg/L, 174 mg/L and 237 mg/L in strains B-5 (*aroG*^*S180F*^)*,* B-6 (*aroG2*) and B-7 (*aroF*). In strains with modified AroG variants, even though the substituted amino-acid residues reside within an identical structural domain, A40926B0 titers differed substantially following heterologous overexpression of each mutated AroG protein. The qRT-PCR analysis was performed to compare the transcript levels of *aroG*^*S180F*^, *aroG*^*L175D*^ and *aroG*^*D146N*^ in the corresponding recombinant strains (Fig. 4C). No obvious transcriptional differences were observed among these three mutant genes, suggesting that the divergent A40926B0 titers did not arise from variable expression of the AroG variants. Although the S180F substitution represents a well-characterized feedback-resistant variant, this mutation unexpectedly lowered A40926B0 biosynthesis to 237 mg/L relative to the parental strain B-1 (400 mg/L). Structural studies have demonstrated that Ser180 resides within the allosteric regulatory site, and substitution of this residue with bulky phenylalanine may disrupt dynamic coupling between the regulatory and catalytic domains [41]. In contrast, the L175D substitution alleviates feedback-mediated inhibition while enhancing specific activity from 2.70 to 4.46 U/mg [39]; previous work has also shown that L175D outperforms S180F for aromatic-compound biosynthesis in yeast-based systems [42]. Similarly, the D146 N substitution confers strong feedback-resistance without impairing catalytic activity [38]. All of these mutated residues lie on the periphery of the phenylalanine-binding pocket, where they introduce steric hindrance against inhibitor binding while preserving intact catalytic machinery [38,39]. Therefore, the divergent A40926B0 titers across these three mutants suggest that distinct physic-chemical features of substituted residues differentially modulate AroG's catalytic efficiency, substrate-binding affinity and structural stability. This process further remodels metabolic flux throughout the shikimate pathway and ultimately shapes A40926B0 biosynthesis. The sharp decline in A40926B0 titers for strains B-6 (*aroG2*, 174 mg/L) and B-7 (*aroF*, 237 mg/L), relative to the parental strain B-1 (400 mg/L), arises from intensified feedback inhibition along the shikimate pathway. As the rate-limiting enzyme of this pathway, DAHP synthase undergoes potent inhibition by L-Tyr and L-Phe [36]. Overexpression of native DAHP synthases elevates intracellular levels of these two aromatic amino acids; accumulated amino-acid molecules then suppress enzyme activity and reduce metabolic flux along the shikimate pathway. This inhibitory effect substantially impairs A40926B0 biosynthesis, given that its four critical aromatic precursors (Hpg, Dpg, L-Tyr and β-Ht) originate from this pathway. Decreased DAHP-synthase activity in turn diminishes intracellular pools of these four precursors and imposes a combined negative effect on heptapeptide assembly. Taken together, these findings further verify that expressing feedback-resistant DAHP-synthase variants constitutes an effective strategy for boosting A40926B0 titers. For the strains overexpressing *aroG*^*D146N*^ or *aroG*^*L175D*^, the best two producers were obtained. To further enhance A40926B0 production, the aforementioned two mutations were jointly introduced into the *aroG* gene to generate *aroG*^*fbr*^. To enable iterative genome editing, we inserted the *aroG*^*fbr*^ gene downstream of ORF7—which lies adjacent to the *dbv* biosynthetic-gene cluster—in *N. gerenzanensis* strain B-1 using the CRISPR-AsCas12f1 system (Fig. S4). Bioinformatic analyses reveal that ORF7 encodes a functionally unannotated hypothetical protein with no roles in biosynthesis, regulation or resistance associated with the *dbv* cluster. Inserting the expression-cassette downstream of this locus will not interfere with native expression of all *dbv* genes. This targeted-insertion strategy places the rate-limiting enzyme-encoding gene in close proximity to the biosynthetic gene cluster, thereby promoting efficient substrate channeling and coordinated pathway operation [43]. The resulting strain B-8 (*aroG*^*fbr*^) showed the highest A40926B0 (641 mg/L) (Fig. 4B), representing a significant improvement over the single-mutation overexpression strains. This combinatorial mutation strategy is highly effective in greatly relieves the feedback inhibition of DAHP synthase (AroG) by aromatic amino acids, which constitutes a major rate-limiting step in the shikimate pathway (Fig. 4B). Previous studies have demonstrated that single-point mutations individually confer partial feedback resistance to AroG, while combining these two substitutions synergistically boosts enzyme activity and carbon flux through the shikimate pathway [44]. This pattern is consistent with our findings that *aroG*^*fbr*^ (harboring both D146 N and L175D substitutions) outperforms constructs bearing only a single mutation. Additionally, Bongaerts emphasized that optimizing the shikimate pathway through engineering feedback-resistant AroG is a core strategy for enhancing the production of secondary metabolites dependent on aromatic precursors [45], further supporting the rationality of our mutation design.

Beyond the modification of DAHP synthase, chorismate mutase, another key enzyme in the shikimate pathway, is also tightly subject to feedback inhibition by L-Tyr and L-Phe [46,47]. Based on our earlier hypothesis that A40926B0 biosynthesis is limited by supplies of shikimate-derived 4-HPP and L-Tyr, we reasoned that relieving feedback inhibition of chorismate mutase would boost production of these two critical precursors. To further expand the intracellular 4-HPP and L-Tyr pool in strain B-8, the codon-optimized *tyrA*^*fbr*^ (M53I and A354V) [48], encoding a feedback-resistant chorismate mutase/prephenate dehydrogenase from *Escherichia coli* was also inserted into the downstream of *ORF7* gene neighboring to A40926B0 biosynthetic gene cluster. The resulting recombinant strain B-9 (*aroG*^*fbr*^ *tyrA*^*fbr*^) achieved a prominent A40926B0 titer of 810 mg/L (Fig. 4B, Fig. S4).

Enhancing the expression of *tyrA*^*fbr*^ facilitates the conversion of chorismate to 4-HPP, a critical intermediate in l-tyrosine biosynthesis that connects the shikimate pathway to aromatic amino acid formation, thereby promoting the de novo synthesis of l-tyrosine. The *E. coli*-derived *tyrA*^*fbr*^ (M53I + A354V) used in this study was codon-optimized to match the codon-usage bias of *N. gerenzanensis* for efficient heterologous expression. This mutant enzyme has previously been employed in yeast to boost hydroxytyrosol and salidroside titers, confirming its robust catalytic performance across distinct microbial chassis [49]. Notably, the *tyrA*^*fbr*^-mediated enhancement of l-tyrosine biosynthesis not only directly supplies precursors for A40926B0 assembly but also indirectly modulates the metabolic flux of downstream phenolic acids, which is consistent with previous findings that overexpression of *tyrA*^*fbr*^ promotes the production of caffeic acid and p-coumaric acid [50,51]. These phenolic acids, as derivatives of aromatic amino acids, share the same shikimate pathway origin as the amino acid precursors of A40926B0, indicating that *tyrA*^*fbr*^ overexpression effectively amplifies the overall metabolic flux through the shikimate pathway rather than merely redirecting carbon flow to l-tyrosine alone. Furthermore, the feedback-resistant property of *tyrA*^*fbr*^ is critical for maintaining stable l-tyrosine production and improving A40926B0 yield, whereas overexpression of the native chorismate mutase (*aroH*) in *N. gerenzanensis* led to a decrease in A40926B0 production (B-10, 478.5 mg/L, Fig. 4B). This is consistent with reports in *Escherichia coli* and *Pichia pastoris*, where expression of feedback-resistant *tyrA* variants significantly elevated intracellular l-tyrosine levels and promoted the production of aromatic secondary metabolites [52,53]. Although the prominent rise in A40926B0 titer observed in our present study preliminarily validates the function of this heterologous enzyme inside N. *gerenzanensis*, dedicated in vivo and in vitro characterizations remain absent. We will carry out systematic experiments in follow-up work to fully define its catalytic properties and regulatory roles within the host-wide metabolic network.

As a branching pathway for l-tyrosine synthesis, prephenate dehydratase (*pheA*) catalyzes the conversion of prephenate to L-Phe [36]. To redirect the metabolic flux in the shikimate pathway toward L-Tyr, the *pheA* gene was knocked out by using the CRISPR-AsCas12f1 system. Compared with strain B-1, strain B-11 (*aroG*^*fbr*^ *tyrA*^*fbr*^*ΔpheA*) produced 892 mg/L of A40926B0, representing a 2.23-fold increase in A40926B0 yield (Fig. 4b, Fig. S2, Fig. S4). This significant yield improvement highlights the synergistic effect of combining feedback-resistant enzyme overexpression (*aroG*^*fbr*^ and *tyrA*^*fbr*^) with *pheA* knockout, which collectively optimizes the metabolic flux distribution in the shikimate pathway and L-Tyr biosynthetic branch by eliminating the competitive consumption of prephenate by the L-Phe biosynthetic pathway, ensuring more prephenate is available for *tyrA*^*fbr*^-catalyzed conversion to L-Tyr and thereby improving the production of L-Tyr-dependent aromatic secondary metabolites, and by reducing L-Phe accumulation, which may alleviate the feedback inhibition of DAHP synthase and chorismate mutase (both sensitive to L-Phe-mediated feedback regulation) [46,47]. This result demonstrates that the deletion of the *pheA* gene and the redirection of metabolic flux in the shikimate pathway toward L-Tyr ultimately promote the production of A40926B0.

### 3.4 Promoter enhancement of the fatty acid metabolic pathway to boost A40926B0 synthesis

**Fig. 5:**
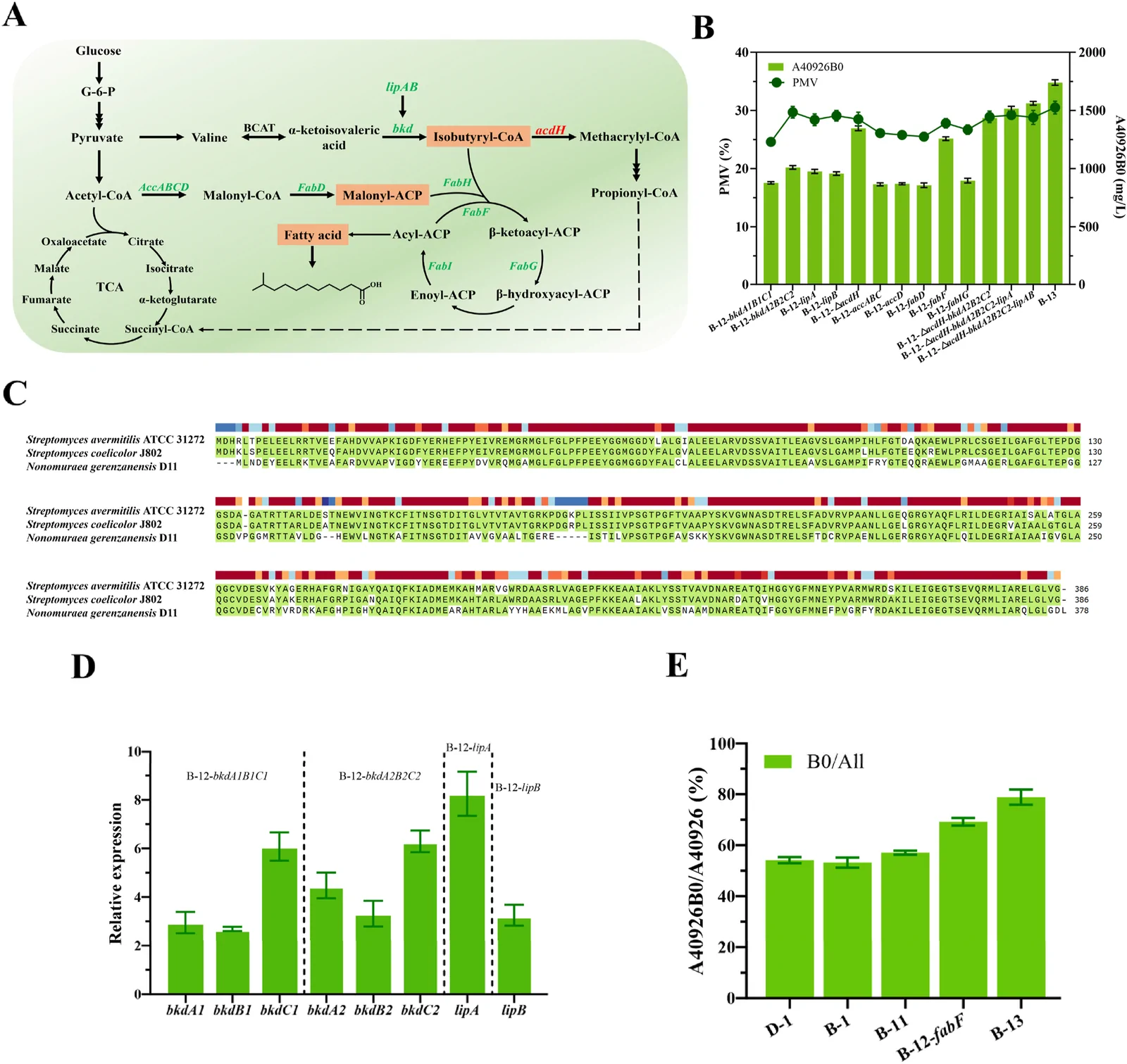
Engineering of the branched-chain fatty acid (BCFA) pathway to enhance A40926B0 biosynthesis. (A) Schematic overview of the BCFA biosynthetic pathway for isododecanoyl side-chain precursor supply in *N. gerenzanensis*. Glucose is catabolized to pyruvate, which enters the TCA cycle or is converted to acetyl-CoA. Acetyl-CoA is carboxylated to malonyl-CoA by acetyl-CoA carboxylase (encoded by *accABCD*). Valine is transaminated to α-ketoisovaleric acid by branched-chain amino acid transaminase (BCAT), then converted to isobutyryl-CoA by the branched-chain α-keto acid dehydrogenase complex (encoded by *bkd*) and lipoyltransferase (encoded by *lipAB*). Isobutyryl-CoA is catabolized to methacrylyl-CoA by acyl-CoA dehydrogenase (encoded by *acdH*), and further to propionyl-CoA. Malonyl-CoA is converted to malonyl-ACP by malonyl-CoA:ACP transacylase (encoded by *FabD*). Fatty acid elongation proceeds via the type II fatty acid synthase (FASII) cycle: β-ketoacyl-ACP synthase III (*fabH*) condenses isobutyryl-CoA with malonyl-ACP to form β-ketoacyl-ACP, which is further elongated by β-ketoacyl-ACP synthase I/II (*fabF*), β-ketoacyl-ACP reductase (*fabG*), enoyl-ACP reductase (*fabI*), and other core FASII enzymes. The resulting acyl-ACP is converted to the isododecanoyl fatty acid side-chain precursor required for A40926B0 biosynthesis. (B) A40926B0 titers (green bars, right y-axis) and packed mycelial volume (PMV, green circles, left y-axis) of strains engineered with BCFA pathway modifications (B-12-A1B1C1 to B-13). Data are presented as mean ± SD (n = 3). (C) Multiple sequence alignment of AcdH homologs from *N*. *gerenzanensis* D11, *Streptomyces coelicolor* J802, and *Streptomyces avermitilis* ATCC 31272. (D) Comparison of A40926B0/A40926 congeners ratio among D-1, B-1, B-11, B-12-*fabF* and B-13 in fermentation medium. Data are presented as mean ± SD (n = 3). (E) Relative transcription levels of *bkdA1B1C1*, *bkdA2B2C2*, *lipA* and *lipB* in the parental strain B-11 and derived engineered strains. qRT-PCR analysis was performed using RNA samples collected from 72 h cultures in fermentation medium. The transcript level of each corresponding gene in parental strain B-11 was arbitrarily assigned a value of 1. The transcription level of *hrdB* was assayed as an internal control. Data are presented as mean ± SD (n = 3).

In addition to the heptapeptide backbone, the branched isododecanoyl side chain represents another key structural moiety of A40926B0. This fatty acyl chain is derived from the branched-chain fatty acid (BCFA) biosynthetic pathway, which belongs to cellular primary metabolism [19,20]. Insufficient expression of genes involved in this pathway often acts as a rate-limiting step that restricts the efficient biosynthesis of the target product. Accordingly, enhancing the intracellular supply of branched-chain fatty acids is considered an effective strategy to improve A40926B0 production. Such metabolic engineering strategies mainly involve three key modules (Fig. 5A): (1) the conversion of valine to isobutyryl-CoA catalyzed by branched-chain amino acid aminotransferase (BCAT) and the branched-chain α-keto acid dehydrogenase (BKD) complex; (2) the transformation of acetyl-CoA to malonyl-ACP mediated by the acetyl-CoA carboxylase (ACC) complex and malonyl-CoA:ACP transacylase (FabD); and (3) the cyclic elongation reaction of BCFA biosynthesis driven by the fatty acid synthase complex.

In the catabolic pathway of BCFAs, the *bkd* gene cluster includes the *bkdA*, *bkdB* and *bkdC* genes that encode the E1α, E1β and E2 subunits of the BKD complex. The BKD complex mediates the oxidative decarboxylation of α-keto acid intermediates derived from branched-chain amino acids (BCAAs), generating the corresponding acyl-CoA derivatives that serve as key precursors for BCFAs biosynthesis. As previously reported, two alleles of the *bkd* gene clusters are located in *N. gerenzanensis* [19]. To enhance the isobutyryl-CoA metabolic pathway, promoter engineering of the *bkd* gene cluster was performed to upregulate the expression of its associated genes. Moreover, insufficient lipoylation mediated by LipAB has been reported to limit BCFA production. In this study, the native promoters of the *bkdA1B1C1*, *bkdA2B2C2*, *LipA* and *LipB* genes were replaced with the strong constitutive promoter *gapdh* [18,54] using the CRISPR/AsCas12f1 system. The *gapdh* promoter has been extensively applied to achieve stable and high-level gene overexpression in actinomycetes without triggering excessive metabolic burdens [31,55]. In comparison with widely used streptomycete-derived promoters, including ermE*p and kasO*, the *gapdh* promoter displays markedly stronger transcriptional activity in *N. gerenzanensis* [18]. The exogenous promoters were integrated into the target chromosomal loci via the homology-directed repair pathway, enabling the precise substitution of native promoters. Diagnostic PCR indicated that the exogenous promoters were integrated into the chromosome of host strain B-11 (Fig. S5). Quantitative real-time PCR (qRT-PCR) analysis confirmed that all target genes exhibited significant upregulation at the transcriptional level in these engineered strains (Fig. 5D). The resultant mutants were designated as B-12-*bkdA1B1C1*, B-12-*bkdA2B2C2*, B-12-*lipA* and B-12-*lipB*, respectively. As shown in Fig. 5B, A40926B0 production reached 1010, 976, 957 mg/L in strain B-12-*bkdA2B2C2*, B-12-*lipA* and B-12-*lipB*. However, overexpression of *bkdA1B1C1* showed no significant effect on the production yield of A40926B0. These results suggest evident functional divergence between the two paralogous *bkd* clusters in *N. gerenzanensis*. This phenomenon frequently occurs among actinomycetes that carry duplicated *bkd* operons to provide precursors for branched-chain fatty-acid biosynthesis. The qRT-PCR results (Fig. 5D) revealed substantial transcriptional up-regulation of *bkdA2B2C2* following *gapdh* promoter replacement, and this elevated transcription markedly promoted A40926B0 biosynthesis. In contrast, transcriptional up-regulation of *bkdA1B1C1* yielded no obvious improvement in product titer. These findings support the notion that *bkdA1B1C1* is primarily dedicated to maintaining the basal BCFA pool for vegetative growth rather than supplying precursors for secondary metabolism, whereas *bkdA2B2C2* specifically furnishes isobutyryl-CoA for secondary-metabolic and lipoglycopeptide biosynthesis. This observation aligns with previously reported distinct physiological functions of paralogous dehydrogenase clusters in *Streptomyces* species [56]. Such cluster-specific specialization highlights the necessity of selecting functionally relevant targets for precise metabolic-engineering strategies. Meanwhile, the pronounced rise in A40926B0 titers after overexpressing *lipA* and *lipB* confirms that lipoylation acts as an essential post-translational modification required for full catalytic activity of the BKD complex. The E2 subunit of BKD requires covalently bound lipoic acid as a prosthetic group to execute oxidative decarboxylation. Insufficient lipoylation is regarded as a rate-limiting step for supplying branched-chain acyl-CoA precursors [57]. Our results demonstrate that co-enhancement of both the BKD catalytic machinery and its lipoylation apparatus effectively alleviate precursor limitation for A40926B0 biosynthesis.

Furthermore, acyl-CoA dehydrogenase (AcdH) acts as a key catabolic enzyme governing the intracellular pool of branched-chain acyl-CoAs. In *Streptomyces* species, AcdH catalyzes the irreversible dehydrogenation of isobutyryl-CoA to methacrylyl-CoA, thus directing isobutyryl-CoA flux toward degradation and reducing precursor availability for lipoglycopeptide biosynthesis [58,59]. In vitro assays have confirmed that Streptomyces-derived AcdH exhibits pronounced substrate specificity: it displays high catalytic activity toward branched-chain isobutyryl-CoA but low activity against linear butyryl-CoA. Mutant experiments in *Streptomyces avermitilis* further validated that although AcdH catalyzes oxidation of n-butyryl-CoA in vitro, it barely participates in n-butyryl-CoA metabolism in vivo; instead, it preferentially converts isobutyryl-CoA into methacrylyl-CoA inside cells [58]. BLAST homology alignment identified a single *acdH* ortholog in *N. gerenzanensis* with high sequence similarity to functionally verified *acdH* from *S. coelicolor* and *S. avermitilis* (Fig. 5C). It shares 74.02% sequence identity with AcdH from *S. avermitilis* (E-value = 0.0) and 73.49% identity with the counterpart from *S. coelicolor* (E-value = 0.0), suggesting a conserved function in isobutyryl-CoA catabolism.

To block isobutyryl-CoA catabolism and further elevate its intracellular pool for A40926B0 biosynthesis, the *acdH* gene was disrupted in strain B-11 to generate mutant B-12-Δ*acdH* (Fig. S5). As shown in Fig. 5B, inactivation of *acdH* significantly increased A40926B0 production to 1347 mg/L, an improvement of approximately 51%, which was remarkably higher than that of the parental strain. This result demonstrates that eliminating the isobutyryl-CoA catabolic branch effectively redirects metabolic flux toward precursor supply rather than degradation. To assess potential cytotoxicity caused by intracellular isobutyryl-CoA buildup, we monitored cell growth by determining PMV at multiple time points during fermentation. The resulting growth curves demonstrated that the Δ*acdH* mutant and its parental strain displayed equivalent growth rates and peak PMV values throughout cultivation (Fig. S6). No evident growth retardation or phenotypic defects were observed in this mutant, confirming that accumulated isobutyryl-CoA exerts no negative effects on cell growth. To our knowledge, this is the first report demonstrating that disruption of isobutyryl-CoA catabolism via *acdH* deletion can significantly enhance the biosynthesis of A40926B0 in *N. gerenzanensis*. Similar strategies have been successfully applied in *Streptomyces* to boost polyketide and fatty acid-derived metabolites by blocking competing catabolic pathways of key precursors [60,61]. Collectively, these results confirm that isobutyryl-CoA supply constitutes a critical rate-limiting bottleneck for A40926B0 biosynthesis, and blocking its catabolic flux represents a powerful strategy for systematically optimizing precursor supply to enhance the industrial production of the dalbavancin precursor A40926B0 in actinomycetes.

The conversion of acetyl-CoA to malonyl-CoA catalyzed by the ACC complex represents the key rate-limiting step in fatty acid biosynthesis. The ACC complex consists of four subunits, namely α-carboxytransferase (*accA*), β-carboxytransferase (*accD*), biotin carboxyl carrier protein (*accB*), and biotin carboxylase (*accC*). Subsequently, the malonyl group is transferred to acyl carrier protein (ACP) catalyzed by FabD, yielding malonyl-ACP, which serves as the direct substrate for fatty acyl chain elongation. Strengthening ACC and FabD activity has been widely used to increase malonyl-ACP supply and boost the biosynthesis of fatty acid-derived metabolites [24,62,63]. To expand the intracellular malonyl-ACP pool in strain B-11, the native promoters of *accABC*, *accD* and *fabD* were individually replaced with the strong promoter *gapdh* to generate strain B-12-*accABC*, B-12-*accD* and B-12-*fabD*, respectively (Fig. 5). However, compared with the parental strain B-11, overexpression of *accABC*, *accD* or *fabD* exerted no significant promoting effect on A40926B0 titer (Fig. 5B). This unexpected result indicates that malonyl-CoA supply as well as its transfer to ACP is not a rate-limiting step in strain B-11. The endogenous expression levels of *accABC* and *fabD* genes are likely already sufficient to meet the metabolic demand for fatty acid chain elongation, and further enhancement cannot provide additional benefits for A40926B0 biosynthesis. Similar phenomena have been reported in *Bacillus* and *Streptomyces* strains, where overexpression of *accABC* and *fabD* showed no obvious improvement in lipoglycopeptide or polyketide production due to sufficient precursor supply [22,64].

To enhance BCFA flux and precursor supply for A40926B0 production, the native promoters of the *fabF* and *fabIG* were individually replaced with the strong promoter *gapdh* to obtain strain B-12-*fabF* and B-12-*fabIG*. Fermentation results showed that *fabF* overexpression resulted in a remarkable increase in A40926B0 production to 1258 mg/L (Fig. S2), whereas *fabIG* overexpression exerted no significant effect. As the key enzyme responsible for carbon-carbon bond formation during the fatty acid chain elongation cycle, β-ketoacyl-ACP synthase FabF was verified as a critical bottleneck for the BCFA biosynthetic pathway in the background of strain B-11. FabF determines the efficiency of fatty acyl chain extension, and its insufficient activity often restricts the biosynthesis of branched-chain fatty acids [65,66]. Our results further confirm that FabF plays a dominant role in regulating BCFA flux for A40926B0 biosynthesis.

Furthermore, the native promoters of *bkdA2B2C2*, *LipA*, *LipB* and *fabF* were sequentially replaced with the strong promoter *gapdh* in the *acdH*-knockout strain B-12-Δ*acdH* to construct the final engineered strain B-13. Fermentation-based assays of sequentially engineered strains yielded A40926B0 titers of 1435 mg/L for B-12-Δ*acdH*-*bkdA2B2C2*, 1515 mg/L for B-12-Δ*acdH*-*bkdA2B2C2*-*lipA*, 1562 mg/L for B-12-Δ*acdH*-*bkdA2B2C2*-*lipAB*, and 1740 mg/L for B-13 (Fig. 5B, Fig. S2). Systematic comparisons against the parental strain B-11 revealed that *acdH* knockout and promoter substitution of *fabF* contributed the most pronounced improvements in antibiotic titers. Strain B-13 achieved a 1.1-fold increase in A40926B0 production compared with B-11. This remarkable improvement indicates that combinatorial engineering targeting isobutyryl-CoA supply, lipoylation, fatty acid elongation and catabolic flux blockage synergistically relieves multiple metabolic bottlenecks. Such combinatorial metabolic engineering strategies have proven highly effective for enhancing the production of complex natural products in actinomycetes [19,67]. We assessed the genetic stability of strain B-13 via seven consecutive rounds of subculturing. The A40926B0 titers fluctuated between 1685 and 1782 mg/L across subcultures. These results demonstrated that this CRISPR-engineered strain possesses favorable genetic stability with negligible genomic variations, satisfying the stability criteria for industrial-scale fermentation (Fig. S7).

We further analyzed the relative proportion of A40926B0 within total lipoglycopeptide mixtures for representative strains (Fig. 5E). Differences among various A40926 congeners primarily arise from variations in branched-chain and straight-chain fatty-acyl groups. Boosting biosynthetic flux toward the branched-chain fatty acids required for B0 formation markedly increased the fraction of the target B0 component. By contrast, strain B-11 exhibited only a modest rise in B0 fraction after shikimate-related modifications. Gratifyingly, the final engineered strain B-13 achieved a B0 proportion of 78.89%, satisfying the FDA industrial criterion (B0 ≥75%) for the dalbavancin precursor.

### 3.5 Batch culture of B-13 in a 50 L fermenter for A40926B0 production

**Fig. 6:**
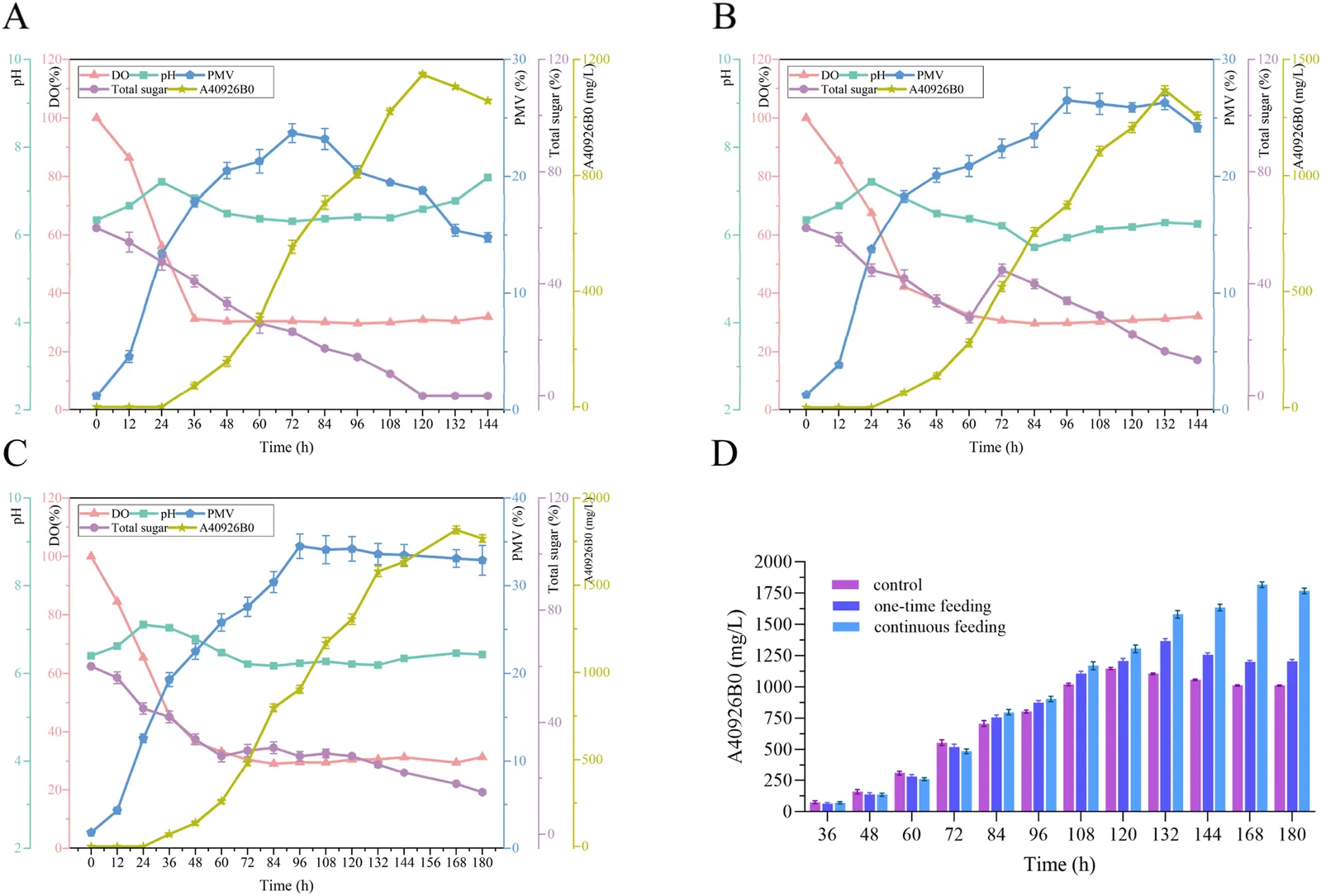
Fermentation performance of engineered strain *N. gerenzanensis* B-13 under different fed-batch strategies in a 50-L fermenter. (A) Batch fermentation profile without feeding. (B) Fermentation profile under the one-time feeding strategy. (C) Fermentation profile under the continuous feeding strategy. (D) Comparison of A40926B0 titers under three different fermentation strategies. Data are presented as mean ± SD (n = 3).

To evaluate the fermentation performance of the engineered strain *N. gerenzanensis* B-13, scale-up cultivation was carried out in a 50 L fermenter. As shown in Fig. 6A, total-sugar concentration decreased drastically from 60 g/L to 33 g/L during the initial 0–48-h fermentation period alongside rapid mycelial proliferation, and the carbon source was completely depleted by 120 h. PMV rose steadily in the early fermentation stage and peaked at 23.7% at 72 h. Over the entire fermentation cycle, pH displayed a three-phase pattern: an initial rise, a subsequent drop to a steady range of 6.3–6.4, and a late-stage rebound. Strain B-13 initiated A40926B0 biosynthesis at 36 h. The maximum titer of 1147.6 mg/L was achieved at 120 h, after which the antibiotic content declined gradually until fermentation ended. Complete depletion of carbon sources at 120 h terminates the supply of carbon precursors, thereby blocking de novo A40926B0 biosynthesis. Furthermore, carbon limitation triggers mycelial autolysis, and hydrolases released from autolyzed cells may slightly degrade the target lipoglycopeptide. Meanwhile, persistent carbon scarcity gradually elevates the fermentation broth pH to nearly 7.5 by 144 h, disrupting the optimal pH for antibiotic synthesis. Collectively, carbon source deficiency in the late fermentation stage is the primary factor responsible for the drop in A40926B0 titer. Accordingly, two maltodextrin-based fed-batch strategies were implemented to improve A40926B0 production: one-time feeding and continuous feeding, both aimed at alleviating carbon limitation in the late fermentation phase. Fig. 6B and C shows the time-course profiles of A40926B0 titer under these two feeding strategies, respectively.

In the one-time feeding strategy, a total of 900 mL maltodextrin solution was supplemented to the 50-L fermenter at 72 h. Relative to the unfed batch control, this single carbon supplementation effectively facilitated mycelial growth. The PMV increased steadily and peaked at 26.5% at 96 h. Nevertheless, this feeding mode induced distinct pH fluctuations during the 72–96 h cultivation period. Notably, the improved biomass accumulation still sustained continuous A40926B0 biosynthesis in the late fermentation stage. The maximum A40926B0 titer finally increased to 1368 mg/L at 132 h. For the continuous feeding strategy, a total of 1200 mL maltodextrin solution was supplemented to the 50-L fermenter from 72 to 120 h. This feeding approach also facilitated mycelial growth, with PMV peaking at 34.5% at 96 h and remaining relatively high throughout the late fermentation stage. Furthermore, continuous feeding maintained a considerably more stable pH during the late fermentation phase. The sustained high PMV and stable pH microenvironment substantially extended the duration of active antibiotic biosynthesis. These comparative results indicated that the one-time addition of excess maltodextrin caused pH drastic fluctuations, thereby inhibiting A40926B0 biosynthesis. In contrast, continuous feeding maintained much more stable pH and higher PMV levels and significantly improved the production of A40926B0. Therefore, continuous feeding is more suitable for A40926B0 production in the 50-L fermenter. The A40926B0 titer reached 1817 mg/L at 168 h (Fig. 6C and D), representing the highest titer and productivity reported to date (Table 1). Considering variations in fermentation scales, media compositions, and feeding strategies across studies, we further cultivated strain B-13 following the IPB-9 and OE protocols, which yielded shake-flask titers of 1013.3 mg/L and 987.5 mg/L, respectively. Overall, the relatively high fermentation yield obtained in this study contributes to reducing production costs, which is of great significance for industrial application.

**Table 1:** A40926B0 production by different *N. gerenzanensis* strains.

Strains	methods	Reported production (mg/L)	References
*N. gerenzanensis*			
B-13	targeted modifications of precursor-supply-related genes	1740 in shake flask, 1817 in 50 L fermenter	This study
IPB-9	Redirection of acyl donor metabolic flux	507.3 in shake flask, 702.4 in 2 L fermenter	[19]
OE	Reshaping of the regulatory network of A40926B0 biosynthesis	192 in shake flask	[20]

## 4 Conclusion

Here, we present the first application of the miniature CRISPR/AsCas12f1 nuclease for efficient genetic manipulation in *N. gerenzanensis*, coupled with pathway-specific metabolic engineering to unlock the biosynthetic potential of A40926B0. The CRISPR/AsCas12f1 system, with its compact size and inherently low toxicity, overcomes the limitations of conventional SpCas9-based tools in this rare actinomycete, enabling rapid and precise genome editing including gene knockout, promoter replacement, and iterative metabolic engineering. Systematic investigation of precursor-supply-related genes revealed that aromatic amino acid and branched-chain fatty acid availability constitute major bottlenecks in A40926B0 biosynthesis. Engineering of the shikimate pathway through overexpression of feedback-resistant *aroG*^*fbr*^ and *tyrA*^*fbr*^, combined with *pheA* deletion to eliminate competitive flux, substantially enhanced l-tyrosine supply. In the branched-chain fatty acid pathway, promoter-driven upregulation of *bkdA2B2C2*, *LipA*, *LipB* and *fabF*, together with *acdH* knockout to block precursor catabolism, effectively improved isobutyryl-CoA availability. Notably, overexpression of the ACC complex and FabD showed no beneficial effect, indicating that malonyl-CoA supply is not rate-limiting and highlighting the importance of identifying genuine metabolic bottlenecks rather than indiscriminate pathway enhancement. The combinatorially engineered strain produced 1740 mg/L A40926B0 in shake flasks and 1817 mg/L in 50-L fed-batch fermentation, representing the highest titer reported to date. These results demonstrate that rational genetic modifications targeting precursor supply, guided by CRISPR/AsCas12f1-enabled rapid strain construction, provides an effective strategy for enhancing complex lipoglycopeptide antibiotic production. The metabolic engineering framework established herein represents a valuable reference for improving the biosynthesis of other natural products dependent on both amino acid and fatty acid precursors in industrially relevant actinomycetes.

## CRediT authorship contribution statement

**Xiaoru Wang:** Data curation, Formal analysis, Methodology, Writing – original draft. **Hongmei Zhai:** Writing – review & editing. **Dandan Gu:** Writing – review & editing. **Yongchao Wang:** Writing – review & editing. **Jiali Gao:** Software, Visualization. **Luxi Du:** Software, Validation. **Liuxu Zhuo:** Software. **Xiaobing Li:** Methodology, Writing – original draft, Writing – review & editing. **Yu Wang:** Methodology, Writing – review & editing.

## Funding

This work was supported by by the Science Research Project of Hebei Education Department (grant number CXZX2025063).

## Declaration of competing interests

The authors declare that they have no known competing financial interests or personal relationships that could have appeared to influence the work reported in this paper.
