# Supplementary material for: Strategy for enhanced production of A40926B0 in Nonomuraea gerenzanensis using an efficient CRISPR/AsCas12f1 system
Source: Synth Syst Biotechnol. 2026 Aug 24;18:49–62. doi: 10.1016/j.synbio.2026.08.006 (PMC13543061; doi:10.1016/j.synbio.2026.08.006)
Supplement: Supplementary file 1 [file mmc1.docx]

**Table S1. Strains and plasmids used in this study**

| Strain or plasmid | Genotype and description | Source and reference |
| --- | --- | --- |
| ***strains*** |  |  |
| *N. gerenzanensis* D11 | producer of A40926B0 | This study |
| B-1 | D11 *dbv23* gene deletion mutant | This study |
| B-2 | *Δdbv23* harboring pSET152-*gapdh*-*aroF^P148L^*; Apra^r^ | This study |
| B-3 | *Δdbv23* harboring pSET152-*gapdh*-*aroG^D146N^*; Apra^r^ | This study |
| B-4 | *Δdbv23* harboring pSET152-*gapdh*-*aroG^L175D^*; Apra^r^ | This study |
| B-5 | *Δdbv23* harboring pSET152-*gapdh*-*aroG^S180F^*; Apra^r^ | This study |
| B-6 | *Δdbv23* harboring pSET152-*gapdh*-*aroG2*; Apra^r^ | This study |
| B-7 | *Δdbv23* harboring pSET152-*gapdh*-*aroF*; Apra^r^ | This study |
| B-8 | *Δdbv23* harboring *gapdh*-*aroG^fbr^* downstream of ORF7 | This study |
| B-9 | *Δdbv23* harboring *gapdh*-*aroG^fbr^-tyrA^fbr^* downstream of ORF7 | This study |
| B-10 | *Δdbv23* harboring *gapdh*-*aroG^fbr^* downstream of ORF7, and pSET152-*gapdh*- *aroH*; Apra^r^ | This study |
| B-11 | *Δdbv23ΔpheA* harboring *gapdh*-*aroG^fbr^-tyrA^fbr^* downstream of ORF7 | This study |
| B-12-bkdA1B1C1 | *Δdbv23ΔpheA* with *bkdA1B1C1* promoter replaced by *gapdh* promoter, harboring *gapdh*-*aroG^fbr^-tyrA^fbr^* downstream of ORF7 | This study |
| B-12-bkdA2B2C2 | *Δdbv23ΔpheA* with *bkdA2B2C2* promoter replaced by *gapdh* promoter, harboring *gapdh*-*aroG^fbr^-tyrA^fbr^* downstream of ORF7 | This study |
| B-12-LipA | *Δdbv23ΔpheA* with *lipA* promoter replaced by *gapdh* promoter, harboring *gapdh*-*aroG^fbr^-tyrA^fbr^* downstream of ORF7 | This study |
| B-12-LipB | *Δdbv23ΔpheA* with *lipB* promoter replaced by *gapdh* promoter, harboring *gapdh*-*aroG^fbr^-tyrA^fbr^* downstream of ORF7 | This study |
| B-12-ΔacdH | *Δdbv23ΔpheAΔacdH* harboring *gapdh*-*aroG^fbr^-tyrA^fbr^* downstream of ORF7 | This study |
| B-12-accABC | *Δdbv23ΔpheA* with *accABC* promoter replaced by *gapdh* promoter, harboring *gapdh*-*aroG^fbr^-tyrA^fbr^* downstream of ORF7 | This study |
| B-12-accD | *Δdbv23ΔpheA* with *accD* promoter replaced by *gapdh* promoter, harboring *gapdh*-*aroG^fbr^-tyrA^fb^r* downstream of ORF7 | This study |
| B-12-fabD | *Δdbv23ΔpheA* with *fabD* promoter replaced by *gapdh* promoter, harboring *gapdh*-*aroG^fbr^-tyrA^fbr^* downstream of ORF7 | This study |
| B-12-fabF | *Δdbv23ΔpheA* with *fabF* promoter replaced by *gapdh* promoter, harboring *gapdh*-*aroG^fbr^-tyrA^fbr^* downstream of ORF7 | This study |
| B-12-fabIG | *Δdbv23ΔpheA* with *fabIG* promoter replaced by *gapdh* promoter, harboring *gapdh*-*aroG^fbr^-tyrA^fbr^* downstream of ORF7 | This study |
| B-13 | *Δdbv23ΔpheAΔacdH* with promoters of *bkdA2B2C2, LipA, LipB* and *fabF* replaced by *gapdh* promoter, harboring *gapdh*-*aroG^fbr^-tyrA^fbr^* downstream of ORF7 | This study |
| ***Escherichia coli*** |  |  |
| *E. coli* DH5α | Host for DNA cloning | This study |
| *E. coli* ET12567/pUZ8002 | ET12567 containing the non-transmissible RP4 derivative plasmid pUZ8002; helper strain for intergeneric conjugation; | Invitrogen |
| **Plasmids** |  |  |
| pSET152 | *E. coli*-*Streptomyces* shuttle vector, φC31 integrase system, Apr^R^ | [1] |
| pSET153 | Suicide plasmid derived from pSET152 | [2] |
| pSET153-dbv23-1.0kb | Suicide plasmid containing 1.0 kb up/downstream homologous arms of *dbv23* | This study |
| pSET153- dbv23-1.5kb | Suicide plasmid containing 1.5 kb up/downstream homologous arms of *dbv23* | This study |
| pSET153- dbv23-2.0kb | Suicide plasmid containing 2.0 kb up/downstream homologous arms of *dbv23* | This study |
| pSET153- dbv23-2.5kb | Suicide plasmid containing 2.5 kb up/downstream homologous arms of *dbv4* | This study |
| pSET153-dbv4-1.0kb | Suicide plasmid containing 1.0 kb up/downstream homologous arms of *dbv4* | This study |
| pSET153- dbv4-1.5kb | Suicide plasmid containing 1.5 kb up/downstream homologous arms of *dbv4* | This study |
| pSET153- dbv4-2.0kb | Suicide plasmid containing 2.0 kb up/downstream homologous arms of *dbv4* | This study |
| pSET153- dbv4-2.5kb | Suicide plasmid containing 2.5 kb up/downstream homologous arms of *dbv4* | This study |
| pKC1139 | Temperature-sensitive shuttle vector; pSG5 ori; Apr^R^ | [1] |
| pAsCas12f1-T | pUC18 carrying the codon-optimized  AsCas12f1 gene under control of *tipA* and sgRNA scaffold (without the spacer sequence, N20) driven by *PJ23119* | This study |
| pCas9-T | pUC18 carrying the codon-optimized  *SpCas9* gene under control of *tipA* and sgRNA scaffold (without the spacer sequence, N20) driven by *PJ23119* | This study |
| pgapdh-T | pUC18 with promoter *gapdh* | This study |
| permE*p-T | pUC18 with promoter *ermE*p* | This study |
| pCas12f1^-^*^tipA^* | pKC1139 containing AsCas12f1 under control of *tipA* and sgRNA scaffold (without the spacer sequence, N20) driven by *PJ23119* | This study |
| pCas12f1^-^*^gapdh^* | pKC1139 containing AsCas12f1 under control of *gapdh* and sgRNA scaffold (without the spacer sequence, N20) driven by *PJ23119* | This study |
| pCas12f1^-^*^ermE*p^* | pKC1139 containing AsCas12f1 under control of *ermE*p* and sgRNA (without the spacer sequence, N20) scaffold driven by *PJ23119* | This study |
| pCas12f1^-^*^tipA^*-*sg^dbv4^* | pCas12f *^tipA^* with the cloning of the guide sequence targeting *dbv4* (nt 789~812 nt with respect to the start codon of *dbv4*) | This study |
| pCas12f1^-^*^gapdh^*-*sg^dbv4^* | pCas12f *^gapdh^* with the cloning of the guide sequence targeting *dbv4* (nt 789~812 nt with respect to the start codon of *dbv4*) | This study |
| pCas12f1^-^*^ermE*p^*-*sg^dbv4^* | pCas12f *^ermE*p^* with the cloning of the guide sequence targeting *dbv4* (nt 789~812 nt with respect to the start codon of *dbv4*) | This study |
| pCas12f1^-^*^gapdh^*-*sg1* | pCas12f^-^*^gapdh^* with the cloning of the guide sequence targeting *dbv4* (nt 789~812 nt with respect to the start codon of *dbv4*) | This study |
| pCas12f1^-^*^gapdh^*-*sg2* | pCas12f^-^*^gapdh^* with the cloning of the guide sequence targeting *dbv4* (nt 843~866 nt with respect to the start codon of *dbv4*) | This study |
| pCas12f1^-^*^gapdh^*-*sg3* | pCas12f^-^*^gapdh^* with the cloning of the guide sequence targeting *dbv4* (nt 120~143 nt with respect to the start codon of *dbv4*) | This study |
| pCas12f1^-^*^gapdh^*-*sg4* | pCas12f^-^*^gapdh^* with the cloning of the guide sequence targeting *dbv4* (nt 807~830 nt with respect to the start codon of *dbv4*) | This study |
| pCas12f1^-^*^gapdh^*-*sg5* | pCas12f^-^*^gapdh^* with the cloning of the guide sequence targeting *dbv23* (nt 1366~1389 nt with respect to the start codon of *dbv23*) | This study |
| pCas12f1^-^*^gapdh^*-*sg6* | pCas12f^-^*^gapdh^* with the cloning of the guide sequence targeting *dbv23* (nt 964~987 nt with respect to the start codon of *dbv23*) | This study |
| pCas12f1^-^*^gapdh^*-*sg7* | pCas12f^-^*^gapdh^* with the cloning of the guide sequence targeting *dbv23* (nt 1013~1036 nt with respect to the start codon of *dbv23*) | This study |
| pCas12f1^-^*^gapdh^*-*sg1*-HA | pCas12f1^-^*^gapdh^*-*sg1* with the cloning of the  homologous arms (approximately 2.0 kb each) flanking *dbv4* | This study |
| pCas12f1^-^*^gapdh^*-*sg2*-HA | pCas12f1^-^*^gapdh^*-*sg2* with the cloning of the  homologous arms (approximately 2.0 kb each) flanking *dbv4* | This study |
| pCas12f1^-^*^gapdh^*-*sg3*-HA | pCas12f1^-^*^gapdh^*-*sg3* with the cloning of the  homologous arms (approximately 2.0 kb each) flanking *dbv4* | This study |
| pCas12f1^-^*^gapdh^*-*sg4*-HA | pCas12f1^-^*^gapdh^*-*sg4* with the cloning of the  homologous arms (approximately 2.0 kb each) flanking *dbv4* | This study |
| pCas12f1^-^*^gapdh^*-*sg5*-HA | pCas12f1^-^*^gapdh^*-*sg5* with the cloning of the  homologous arms (approximately 2.0 kb each) flanking *dbv23* | This study |
| pCas12f1^-^*^gapdh^*-*sg6*-HA | pCas12f1^-^*^gapdh^*-*sg6* with the cloning of the  homologous arms (approximately 2.0 kb each) flanking *dbv23* | This study |
| pCas12f1^-^*^gapdh^*-*sg7*-HA | pCas12f1^-^*^gapdh^*-*sg7* with the cloning of the  homologous arms (approximately 2.0 kb each) flanking *dbv23* | This study |
| pCas9^-^*^gapdh^* | pKC1139 containing SpCas9 under control of *gapdh* and sgRNA scaffold (without the spacer sequence, N20) driven by *PJ23119* | This study |
| pCas9^-^*^ermE*p^* | pKC1139 containing SpCas9 under control of *ermE*p* and sgRNA scaffold (without the spacer sequence, N20) driven by *PJ23119* | This study |
| pCas9^-^*^tipA^* | pKC1139 containing SpCas9 under control of *tipA* and sgRNA scaffold (without the spacer sequence, N20) driven by *PJ23119* | This study |
| pCas9^-^*^gapdh^-sg^dbv4^* | pCas9^-^*^gapdh^* with the cloning of the guide sequence targeting *dbv4* (nt 614~633 nt with respect to the start codon of *dbv4*) | This study |
| pCas9^-^*^ermE*p^-sg^dbv4^* | pCas9^-^*^ermE*p^* with the cloning of the guide sequence targeting *dbv4* (nt 614~633 nt with respect to the start codon of *dbv4*) | This study |
| pCas9^-^*^tipA^-sg^dbv4^* | pCas9^-^*^tipA^* with the cloning of the guide sequence targeting *dbv4* (nt 614~633 nt with respect to the start codon of *dbv4*) | This study |
| p*aroF^P148L^*-T | pUC18 carrying the codon-optimized  *aroF^P148L^* under *gapdh* promoter | This study |
| p*aroG^D146N^*-T | pUC18 carrying the codon-optimized  *aroG^D146N^* under *gapdh* promoter | This study |
| p*aroG^L175D^*-T | pUC18 carrying the codon-optimized  *aroG^L175D^* under *gapdh* promoter | This study |
| p*aroG^S180F^*-T | pUC18 carrying the codon-optimized  *aroG^S180F^* under *gapdh* promoter | This study |
| p*aroG^fbr^*-T | pUC18 carrying the codon-optimized  *aroG^fbr^* under *gapdh* promoter | This study |
| pSET152-*gapdh*-*aroF^P148L^* | pSET152 derivative containing the *aroF^P148L^* under *gapdh* promoter | This study |
| pSET152-*gapdh*-*aroG^D146N^* | pSET152 derivative containing the *aroG^D146N^* under *gapdh* promoter | This study |
| pSET152-*gapdh*-*aroG^L175D^* | pSET152 derivative containing the *aroG^L175D^* under *gapdh* promoter | This study |
| pSET152-*gapdh*-*aroG^S180F^* | pSET152 derivative containing the *aroG^S180F^* under *gapdh* promoter | This study |
| pSET152-*gapdh*-*aroG2* | pSET152 derivative containing the *aroG2* under *gapdh* promoter | This study |
| pSET152-*gapdh*-*aroF* | pSET152 derivative containing the *aroF* under *gapdh* promoter | This study |
| pCas12f1^-^*^gapdh^*-*aroG^fbr^*-HA*^ORF7^* | plasmid used for inserting *aroG^fbr^* into the downstream of *ORF7* gene | This study |
| pCas12f1^-^*^gapdh^*-*tyrA^fbr^*-HA*^ORF7^* | plasmid used for inserting *tyrA^fbr^* into the downstream of *ORF7* gene | This study |
| pCas12f1^-^*^gapdh^*-*ΔpheA* | plasmid used for deleting *pheA* | This study |
| pCas12f1^-^*^gapdh^*-*gapdh*- *bkdA1B1C1* | plasmid used for replacing the native promoter of *bkdA1B1C1* with *gapdh* | This study |
| pCas12f1^-^*^gapdh^*-*gapdh*- *bkdA2B2C2* | plasmid used for replacing the native promoter of *bkdA2B2C2* with *gapdh* | This study |
| pCas12f1^-^*^gapdh^*-*gapdh*-*LipA* | plasmid used for replacing the native promoter of *LipA* with *gapdh* | This study |
| pCas12f1^-^*^gapdh^*-*gapdh*- *LipB* | plasmid used for replacing the native promoter of *LipB* with *gapdh* | This study |
| pCas12f1^-^*^gapdh^*-*ΔacdH* | plasmid used for deleting *acdH* | This study |
| pCas12f1^-^*^gapdh^*-*gapdh*-*accABC* | plasmid used for replacing the native promoter of *accABC* with *gapdh* | This study |
| pCas12f1^-^*^gapdh^*-*gapdh*-*accD* | plasmid used for replacing the native promoter of *accD* with *gapdh* | This study |
| pCas12f1^-^*^gapdh^*-*gapdh*-*fabD* | plasmid used for replacing the native promoter of *fabD* with *gapdh* | This study |
| pCas12f1^-^*^gapdh^*-*gapdh*-*fabF* | plasmid used for replacing the native promoter of *fabF* with *gapdh* | This study |
| pCas12f1^-^*^gapdh^*-*gapdh-fabIG* | plasmid used for replacing the native promoter of *fabIG* with *gapdh* | This study |

**Table S2. Primers used in this study**

| Primer | Sequence(5’→3’) |
| --- | --- |
| dbv23-U-F1 | tctagagtggtgggggcatgagcgat |
| dbv23-U-R1 | gcgcccgactcgcggcatc |
| dbv23-D-F1 | ccgcgagtcgggcgcctgttcggtgggtgccggg |
| dbv23-D-R1 | ggatcccctgctctcgtactcggcca |
| dbv23-U-F2 | tctagacgtcgcagtcacacgttctct |
| dbv23-D-R2 | ggatccgtcgtacagccgggacacca |
| dbv23-U-F3 | tctagacggcgaccaggtcgtggttgt |
| dbv23-D-R3 | ggatcccaaccggtgggcgatcacca |
| dbv23-U-F4 | tctagacacgcggacggcggagtcat |
| dbv23-D-R4 | ggatcccggagtccgccaccatgaac |
| dbv4-U-F1 | tctagaggagtacggccaggcggcccg |
| dbv4-U-R1 | gcacgagtggatgagccgggcg |
| dbv4-D-F1 | ctcatccactcgtgcctagcctccgattcgattagc |
| dbv4-D-R1 | ggatccggcggaggtgccgatcagacc |
| dbv4-U-F2 | tctagaagaagcaccgggtggacgtgg |
| dbv4-D-R2 | ggatccgtgcggggcgtgcgacacca |
| dbv4-U-F3 | tctagaaggctgctccgtgaccaggtg |
| dbv4-D-R3 | ggatccgcgtccggtcgatcctcgagtt |
| dbv4-U-F4 | tctagagaccgtgctgcgcgtgatgta |
| dbv4-D-R4 | ggatccgccgcatgccgcccctgatc |
| Cas12-F/D | aagcttcacactccacaagctagctc |
| Cas12-F/D | gaattcttacttgatcgactcggcga |
| gapdh-F/R | tctagagctgctccttcggtcggacg |
| gapdh-F/R | catatggcgtatcccctttcagatactcg |
| ermE*p-F/R | tctagaggtaccagcccgacccgagca |
| ermE*p-F/R | catatgggtcctcctgtggagtggtt |
| dbv4sgRNA1F | actagtgggattcgtcggttcagcga |
| dbv4sgRNA1R | aagcttttgcgaatgctatcattgcacacactccacaagctagctc |
| dbv4sgRNA2R | aagcttcgcggagtgccgccgcattgcacactccacaagctagctc |
| dbv4sgRNA3R | aagctttcggcacaaggggagcttcccacactccacaagctagctc |
| dbv4sgRNA4R | aagcttcactccatcgacgggcaggacacactccacaagctagctc |
| dbv4LF | acgacggccagtgccaagctttggtagtgcgccctcgcgag |
| dbv4LR | gcacgagtggatgagccggg |
| dbv4RF | ctcatccactcgtgcctagcctccgattcgattag |
| dbv4RR | agcttgtggagtgtgaagcttacaccatagctcgcgtccgg |
| dbv23sgRNA5F | aagctttccttcaacgcgacctgaggcacactccacaagctagctc |
| dbv23sgRNA6F | aagcttccgagccactggaccggccgcacactccacaagctagctc |
| dbv23sgRNA7F | aagcttgccgccgatcatgatcctcccacactccacaagctagctca |
| dbv23LF | acgacggccagtgccaagcttaggtcgtggttgttcgccgg |
| dbv23LR | gcgcccgactcgcggcatct |
| dbv23RF | agatgccgcgagtcgggcgcctgttcggtgggtgccgggc |
| dbv23RR | agcttgtggagtgtgaagcttgggcgatcaccagggaggta |
| Cas9-F | aagcttctcaaaaaaagcaccgactcgg |
| Cas9-D | gaattctcagtcgccgcccagctggga |
| Cas9-dbv4sgRNA-F | actagtgctctcaccgtgctccaaccgttttagagctagaaatagc |
| Cas9-dbv4sgRNA-R | aagcttctcaaaaaaagcaccgactcgg |
| dbv4-test-F | aggtcatgcgcccgagtacg |
| dbv4-test-R | atttggacacgccggatcgc |
| dbv23-test-F | agaatggccgcctgctgctg |
| dbv23-test-R | ctctccgtcgcccttcagcaa |
| aroF-R | catatgatggtgatcgtgatggggcc |
| aroF-F | ggatcctcaggccgtcgcggccgtctt |
| aroG2-R | catatggtgagcagcaatctagtggat |
| aroG2-F | ggatcctcagccgcggtagagctccg |
| OFR7sgRNA1R | aagcttaagcggcgggcgggcgagcgcacactccacaagctagctc |
| OFR7-LL-1 | acgacggccagtgccaagcttctgatggacccggccatcga |
| OFR7-LR-1 | caatccaaccaggttgtccacc |
| OFR7-RL | gctcatatcgctcttgaagcgg |
| OFR7-RR | agcttgtggagtgtgaagcttcatcgccaaccagaacctgt |
| aroG-R | ccgcaaggccatccacaaga |
| aroG-F | ccttcagctcctcccggagg |
| gadph-aroG^fbr^-F | ggtggacaacctggttggattggctgctccttcggtcggacg |
| aroG^fbr^-R | ccgcttcaagagcgatatgagcttagccgcgccgcgccttga |
| aroG^fbr^-test-F | gctgctccttcggtcggacg |
| aroG^fbr^-test-R | ttagccgcgccgcgccttga |
| OFR7-test1-F | ggtggacaacctggttggattg |
| OFR7-test1-R | ccgcttcaagagcgatatgagc |
| OFR7-LL-2 | acgacggccagtgccaagctttggtgcgcgagcacggcctc |
| OFR7-LR-2 | ttagccgcgccgcgccttga |
| tyrA^fbr^-F | tcaaggcgcggcgcggctaaatggtcgccgagctgaccgc |
| tyrA^fbr^-R | ccgcttcaagagcgatatgagcttactggcggttgtcgttgg |
| tyrA^fbr^-test-F | atggtcgccgagctgaccgc |
| tyrA^fbr^-test-R | ttactggcggttgtcgttgg |
| OFR7-test2-F | tcaaggcgcggcgcggctaa |
| pheAsgRNA-F | actagt attggccgccgatcatgatcctccgggattcgtcggttcagcga |
| pheALF | acgacggccagtgccaagcttctccagcccgtatgagtcgg |
| pheALR | acttacaggctatccgcaggtg |
| pheARF | cacctgcggatagcctgtaagtgccggaagccgagggttcg |
| pheARR | agcttgtggagtgtgaagcttctgaccaggcagtacgacgac |
| pheA-test1-F | tgtccacctgcggatagcctg |
| pheA-test1-R | agacctgatcggcccgaacc |
| pheA-test2-F | atgcccagactcgcctatct |
| pheA-test2-R | tcagacctgaccggccctga |
| pbkdA1B1C1sgRNAF | actagtaacggatggccagacagacggggattcgtcggttcagcga |
| bkdA1B1C1-LF | acgacggccagtgccaagctttacacgcatctggacgtgtgg |
| bkdA1B1C1-LR | cgtccgaccgaaggagcagcgaggacactcactttctgctgg |
| bkdA1B1C1-RF | cgagtatctgaaaggggatacgcatgtccacgaagacgtcggc |
| bkdA1B1C1-RR | agcttgtggagtgtgaagcttcggcggataggggatgtcgaa |
| gapdh-F | gctgctccttcggtcggacg |
| gapdh-R | gcgtatcccctttcagatactcg |
| bkdA1B1C1-test1-R | gctgctccttcggtcggacg |
| bkdA1B1C1-test1-F | cgatgaaggccatcaccacg |
| pbkdA2B2C2sgRNAF | actagtgtaaccccatccgaagtccggggattcgtcggttcagcga |
| bkdA2B2C2-LF | acgacggccagtgccaagcttcttcatccccagctcggcgaa |
| bkdA2B2C2-LR | cgtccgaccgaaggagcagcgcgccactcctgctggtcag |
| bkdA2B2C2-RF | cgagtatctgaaaggggatacgcatgaccatagacgcctcgggag |
| bkdA2B2C2-RR | agcttgtggagtgtgaagcttgtcgagcacgcggtccaggt |
| bkdA2B2C2-test1-R | gctgctccttcggtcggacg |
| bkdA2B2C2-test1-F | ccgtcgccgaagtagacgat |
| plipAsgRNAF | actagtctcctgaaggccgaaagctcgggattcgtcggttcagcga |
| lipA-LF | acgacggccagtgccaagcttgcactacaactccgagccgca |
| lipA-LR | acgacggccagtgccaagctttcaggagatcagcccgaagcc |
| lipA-RF | acgacggccagtgccaagcttatgcgtctctgcgctctcgt |
| lipA-RR | acgacggccagtgccaagcttgagcgcctgagccaggcacc |
| lipA-test1-R | gctgctccttcggtcggacg |
| lipA-test1-F | tcctccagctgctcacgatc |
| plipBsgRNAF | actagtctgaccccctgattacccttgggattcgtcggttcagcga |
| lipB-LF | acgacggccagtgccaagcttgcacgggcgccgtcgtgaag |
| lipB-LR | acgacggccagtgccaagctttcagggggtcagcaaacgag |
| lipB-RF | acgacggccagtgccaagcttatgcgccccatccccgggga |
| lipB-RR | acgacggccagtgccaagcttcccggcgcacgagcgatttca |
| lipB-test1-F | gctgctccttcggtcggacg |
| lipB-test1-R | tggcgcagttgagggcgaag |
| hrdB-F | tgaggaggactccgacctcg |
| hrdB-R | gagctggatgatgcgctgctc |
| bkdA1-F | cacgaagacgtcggcgagg |
| bkdA1-R | ccaccggtacgcctcgc |
| BkdB1-F | tgaccagggacttcggcgag |
| BkdB1-R | tcgaacgccgggtaggc |
| BkdC1-F | gatctgggggaagggctgac |
| BkdC1-R | gcacgacccccgcatacg |
| BkdA2-F | gccctgctcgttcttgaacagg |
| BkdA2-R | catcgaggcgtacacgtaccg |
| BkdB2-F | ggtcgggcaggtagtgctc |
| BkdB2-R | ccggtcaacagcggtttcg |
| BkdC2-F | cacgccgacgttggtgatg |
| BkdC2-R | ggtccccaacatcaaggacgc |
| lipA-F | tactgcgtgatcgtcagcag |
| lipA-R | tcgctcggcgtgatcacgat |
| lipB-F | agttgagggcgaagccgtg |
| lipB-R | acttcggcgtcaccgccag |
| acdHsgRNA-F | actagt aactggatggcctggtagtggggattcgtcggttcagcga |
| acdHLF | acgacggccagtgccaagcttatcgggtcgacgcagtggtc |
| acdHLR | acaggtgtttgccttatgcgc |
| acdHRF | gcgcataaggcaaacacctgtgtctcagcctttcgaaagcgc |
| acdHRR | agcttgtggagtgtgaagcttgaggtgcggctcgacgggca |
| acdH-test-F | atgctcaacgacgagtacgaagaact |
| acdH-test-R | tcacaggtcccccagcccga |
| paccABCsgRNAF | actagtacgtaccggccgaggtcggcgggattcgtcggttcagcga |
| accABC-LF | acgacggccagtgccaagcttcactgatgagagcccactga |
| accABC-LR | acgacggccagtgccaagctttcatgagctgcgctcccacc |
| accABC-RF | acgacggccagtgccaagcttatgaccctcacgctgagcgac |
| accABC-RR | acgacggccagtgccaagctttccacgacctcgcccgacca |
| accABC-test1-R | gctgctccttcggtcggacg |
| accABC-test1-F | gtcctggccatctgcatgag |
| paccDsgRNAF | actagtacgcatgagcgccctggccggggattcgtcggttcagcga |
| accD-LF | acgacggccagtgccaagcttgcgttcctgcgcgacctgga |
| accD-LR | acgacggccagtgccaagctttcaactttcgcgctcctcca |
| accD-RF | acgacggccagtgccaagcttgtgagccgtccggacgcgcg |
| accD-RR | acgacggccagtgccaagcttgatctggcacgtcacgatcc |
| accD-test1-R | gctgctccttcggtcggacg |
| accD-test1-F | ggctccgccaccgtgacgtg |
| pfabDsgRNAF | actagttcctgggtgatttgtaggaagggattcgtcggttcagcga |
| fabD-LF | acgacggccagtgccaagcttaacctgctgcccgcctacct |
| fabD-LR | acgacggccagtgccaagcttttacgttagggcccgccagg |
| fabD-RF | acgacggccagtgccaagcttgtgcttgtactcgtcgctccg |
| fabD-RR | acgacggccagtgccaagctttgcccgtgatctcgttgatgat |
| fabD-test1-R | gctgctccttcggtcggacg |
| fabD-test1-F | gaacgcggccagctgctcca |
| pfabFsgRNAF | actagttgcgcgacgacgcccgtcggtggggattcgtcggttcagcga |
| fabF-LF | acgacggccagtgccaagcttctgctcgtagccgccgcgct |
| fabF-LR | acgacggccagtgccaagcttttagttctggatgaagttgagg |
| fabF-RF | acgacggccagtgccaagcttgtgagtgcgaaccgggtacgt |
| fabF-RR | acgacggccagtgccaagcttgaaacgggaggtccggtgtc |
| fabF-test1-R | gctgctccttcggtcggacg |
| fabF-test1-F | catcgcgtagccgatggcct |
| pfabIGsgRNAF | actagttccgggtggcacaagatcacgggattcgtcggttcagcga |
| fabIG-LF | acgacggccagtgccaagcttcgtccacgccgagcgcttca |
| fabIG-LR | acgacggccagtgccaagcttatgcgccagatcgaccccga |
| fabIG-RF | acgacggccagtgccaagcttatggcacgctctgtactcgt |
| fabIG-RR | acgacggccagtgccaagcttgacggcggtcacgatgtgct |
| fabIG-test1-R | gctgctccttcggtcggacg |
| fabIG-test1-F | aagccgggcgagacgacgtt |

**Fig.S1**

Fig.S1 Mycelial morphology and growth curve of *N. gerenzanensis* D11. (A) Microscopic observation of mycelial structures of strain D11 cultured in four different media (R3, TSB, VSP and YEME). (B) Growth curve of D11 cultured in VSP liquid medium. OD₆₀₀ was measured at time points ranging from 6 h to 48 h, and error bars indicate the standard error of three biological replicates.

**Fig.S2**

Fig.S2 HPLC analysis of A40926B0 in fermentation broths. (i) A40926B0 standard, (ii) D11, (iii) B-1, (iv) B-11, (v) B-12-fabF, (vi) B-13.

**Fig.S3**

Fig.S3 Mismatch tolerance assay of AsCas12f1-mediated genome editing in *N. gerenzanensis.* Top: Alignment of target genomic DNA double strands, with green-shaded PAM and position markers 1–20. Left: Sequences of perfect-match sgRNA (PM) and mismatched sgRNA variants (MM), mismatched bases labeled in color. Right bar chart: Percentage of dbv4 knockout-positive strains after conjugation screening. 100% editing efficiency was only observed in PM group, while no edited strains were obtained for all 1–2 nt mismatch groups, proving low off-target risk of AsCas12f1.

**Fig.S4**

Fig.S4 Schematic diagram of the construction process and PCR verification of the strains. The *aroG^fbr^* and *tyrA^fbr^* genes were sequentially inserted downstream of ORF7, followed by deletion of the *pheA* gene. Two pairs of primers (outer and inner primers) were used for PCR verification at each step. (A) Schematic representation of the genetic manipulation process and the positions of the verification primers. (B) PCR verification of *aroG^fbr^* insertion downstream of ORF7. Lanes 1 (outer primer pair) and 2 (inner primer pair): control strain; Lanes 3 (outer primer pair) and 4 (inner primer pair): the correct recombinant strain (B-8). (C) PCR verification of *tyrA^fbr^* insertion downstream of ORF7. Lanes 1 (outer primer pair) and 2 (inner primer pair): control strain; Lanes 3 (outer primer pair) and 4 (inner primer pair): the correct recombinant strain (B-9). (D) PCR verification of *pheA* gene deletion. Lanes 1 (outer primer pair) and 2 (inner primer pair): control strain; Lanes 3 (outer primer pair) and 4 (inner primer pair): the correct recombinant strain (B-11).

**Fig.S5**

Fig.S5 Colony PCR verification of promoter replacement and gene knockout. For all targets, Lanes 1–2 are wild-type controls, and Lanes 3–4 are verified positive clones (promoter replacement for *bkdA1B1C1*, *bkdA2B2C2*, *lipA*, *lipB*, *accABC*, *accD*, *fabD*, *fabF*, *fabG*; gene knockout for *acdH*).

**Fig.S6**

Fig.S7 Growth curve of *N. gerenzanensis* B-11 and B-12-ΔacdH in fermentation culture.

**Fig.S7**

Fig.S8 A40926B0 fermentation titers of strain B-13 after 1–7 successive subcultures. Data are presented as mean ± standard deviation of biological replicates.

**Supplementary references**

[1]	Bierman M, Logan R, O'Brien K, Seno ET, Rao RN, Schoner BE. Plasmid cloning vectors for the conjugal transfer of DNA from Escherichia coli to Streptomyces spp. Gene 1992;116(1):43–9. <http://doi.org/10.1016/0378-1119(92)90627-2>

[2]	Qian H, Wei W, Chen XA, Mo XT, Ge M, Zhao QW, et al. Strategy for Producing the High-Quality Glycopeptide Antibiotic A82846B in *Amycolatopsis orientalis* Based on the CRISPR-Cas12a System. ACS Synth Biol 2021;10(11):3009–16. <http://doi.org/10.1021/acssynbio.1c00317>
